# Toward On-Demand Polymorphic Transitions of Organic Crystals via Side Chain and Lattice Dynamics Engineering

**DOI:** 10.1021/jacs.4c11289

**Published:** 2024-11-08

**Authors:** Luca Catalano, Rituraj Sharma, Durga Prasad Karothu, Marco Saccone, Oren Elishav, Charles Chen, Navkiran Juneja, Martina Volpi, Rémy Jouclas, Hung-Yang Chen, Jie Liu, Guangfeng Liu, Elumalai Gopi, Christian Ruzié, Nicolas Klimis, Alan R. Kennedy, T. Kyle Vanderlick, Iain McCulloch, Michael T. Ruggiero, Panče Naumov, Guillaume Schweicher, Omer Yaffe, Yves H. Geerts

**Affiliations:** † Laboratoire de Chimie des Polymères, Université Libre de Bruxelles (ULB), 1050 Brussels, Belgium; ‡ Department of Chemistry, University of Rochester, Rochester, New York 14627, United States; § Dynamic Molecular Materials Laboratory, Dipartimento di Scienze della Vita, Università degli Studi di Modena e Reggio Emilia, 41125 Modena, Italy; ∥ Department of Chemical and Biological Physics, Weizmann Institute of Science, 76100 Rehovot, Israel; ⊥ Centre for Scientific and Applied Research (CSAR), IPS Academy, Indore 452012, India; # Smart Materials Lab, New York University Abu Dhabi, PO Box 129188 Abu Dhabi, UAE; ∇ Dipartimento di Scienze e Innovazione Tecnologica, Università del Piemonte Orientale, 15121 Alessandria, Italy; ○ Department of Chemical and Environmental Engineering, Yale University, New Haven, Connecticut 06520, United States; ◆ Department of Chemistry and Centre for Plastic Electronics, Imperial College London, London SW7 2AZ, U.K.; ¶ Department of Physics, University of Warwick, Coventry CV4 7AL, U.K.; †† Jiangsu Key Laboratory of Advanced Catalytic Materials & Technology, School of Petrochemical Engineering, Changzhou University, Changzhou 213164, P. R. China; ‡‡ Ohme, 1050 Brussels, Belgium; §§ Department of Pure and Applied Chemistry, University of Strathclyde, Glasgow G1 1XL, U.K.; ∥∥ Andlinger Center for Energy and the Environment and Department of Electrical and Computer Engineering, Princeton University, Princeton, New Jersey 08544, United States; ⊥⊥ Department of Chemistry, Chemistry Research Laboratory, University of Oxford, Oxford OX1 3TA, U.K.; ## Center for Smart Engineering Materials, New York University Abu Dhabi, PO Box 129188 Abu Dhabi, UAE; ∇∇ Research Center for Environment and Materials, Macedonian Academy of Sciences and Arts, Skopje, MK-1000, Macedonia; ○○ Molecular Design Institute, Department of Chemistry, New York University, New York, New York 10003, United States; ◆◆ International Solvay Institutes of Physics and Chemistry, 1050 Brussels, Belgium

## Abstract

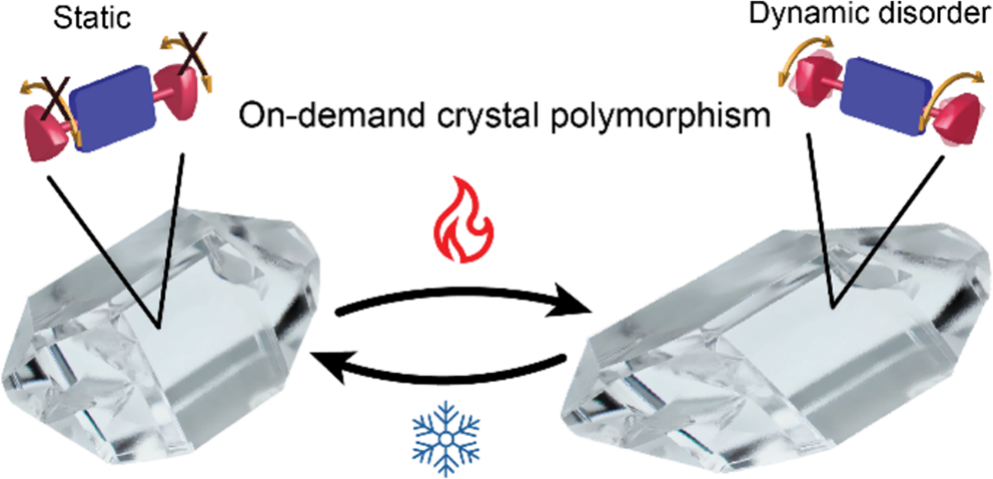

Controlling polymorphism, namely, the occurrence of multiple crystal forms for a given compound, is still an open technological challenge that needs to be addressed for the reliable manufacturing of crystalline functional materials. Here, we devised a series of 13 organic crystals engineered to embody molecular fragments undergoing specific nanoscale motion anticipated to drive cooperative order–disorder phase transitions. By combining polarized optical microscopy coupled with a heating/cooling stage, differential scanning calorimetry, X-ray diffraction, low-frequency Raman spectroscopy, and calculations (density functional theory and molecular dynamics), we proved the occurrence of cooperative transitions in all the crystalline systems, and we demonstrated how both the molecular structure and lattice dynamics play crucial roles in these peculiar solid-to-solid transformations. These results introduce an efficient strategy to design polymorphic molecular crystalline materials endowed with specific molecular-scale lattice and macroscopic dynamics.

## Introduction

Crystalline organic materials have an enormous technological impact on a broad range of fields, such as drug development, pigments, energetic materials, fertilizers, organic electronics, and optoelectronics.^1^ The physicochemical properties of organic crystals are dictated by the chemical identity of the constituent molecular building blocks and by the collective, viz., supramolecular, organization of the molecules within the lattice.^2,3^ Therefore, the precise control of molecular packing is a fundamental requirement for the implementation of such materials in the manufacturing industry. In this context, controlling the commonly observed crystal polymorphism, defined as the occurrence of multiple crystalline phases for a given molecule or molecular assembly, has a paramount importance for the reliable design and synthesis of crystalline materials, but it also poses a formidable challenge due to the rich polymorphic landscape of organic crystals as a direct consequence of the often small differences in lattice energy (≤10 kJ/mol) between the various crystal forms.^4,5^ Solid-state polymorphic transitions induced by external stimuli, e.g., heat, light, and pressure, can be exploited to effectively modulate crystal structures accessing smart and adaptive dynamic crystals.^6,7^ However, even though great advances have been made in predicting crystal structures and polymorphism and understanding polymorphic transition mechanisms,^8,9^ the experimental discovery of new polymorphic crystalline materials and their associated transformations remains a largely serendipitous exercise that relies on crystallographic and chemical intuition.^10^

Cooperative phase transitions are defined as the first-order thermoelastic single-crystal-to-single-crystal reversible transitions that proceed without atomic and/or molecular diffusion.^11,12^ Despite being extensively studied in metals, alloys, and ceramics, reports on cooperative polymorphic transitions of organic crystals are still rare and correlations between the chemical identity of the molecules, the crystal structures, and the transition mechanisms remain unclear and poorly understood.^12,13^ These peculiar transformations are particularly interesting because they are associated with fast and reversible tuning of the physicochemical properties of the materials and macroscopic dynamic effects, such as crystal jumping, rolling, twisting, and changing shape, phenomena that hold potential for advanced applications as sensors, actuators, and switchable and flexible electronic devices.^14,15^ They are generally accompanied by specific molecular rearrangements, namely gliding, conformational changes, and molecular rotation,^12,16^ and they appear to be driven by specific lattice vibrations, as recently reported by some of the authors.^17,18^ Several studies have shown that side-chain engineering of π-conjugated aromatic cores with either rotating bulky side chains like *tert*-butyl groups or alkyl chains results in cooperative phase transitions in the corresponding crystals.^19−23^ However, no generally applicable molecular strategy has been reported so far.

**Figure 1:**
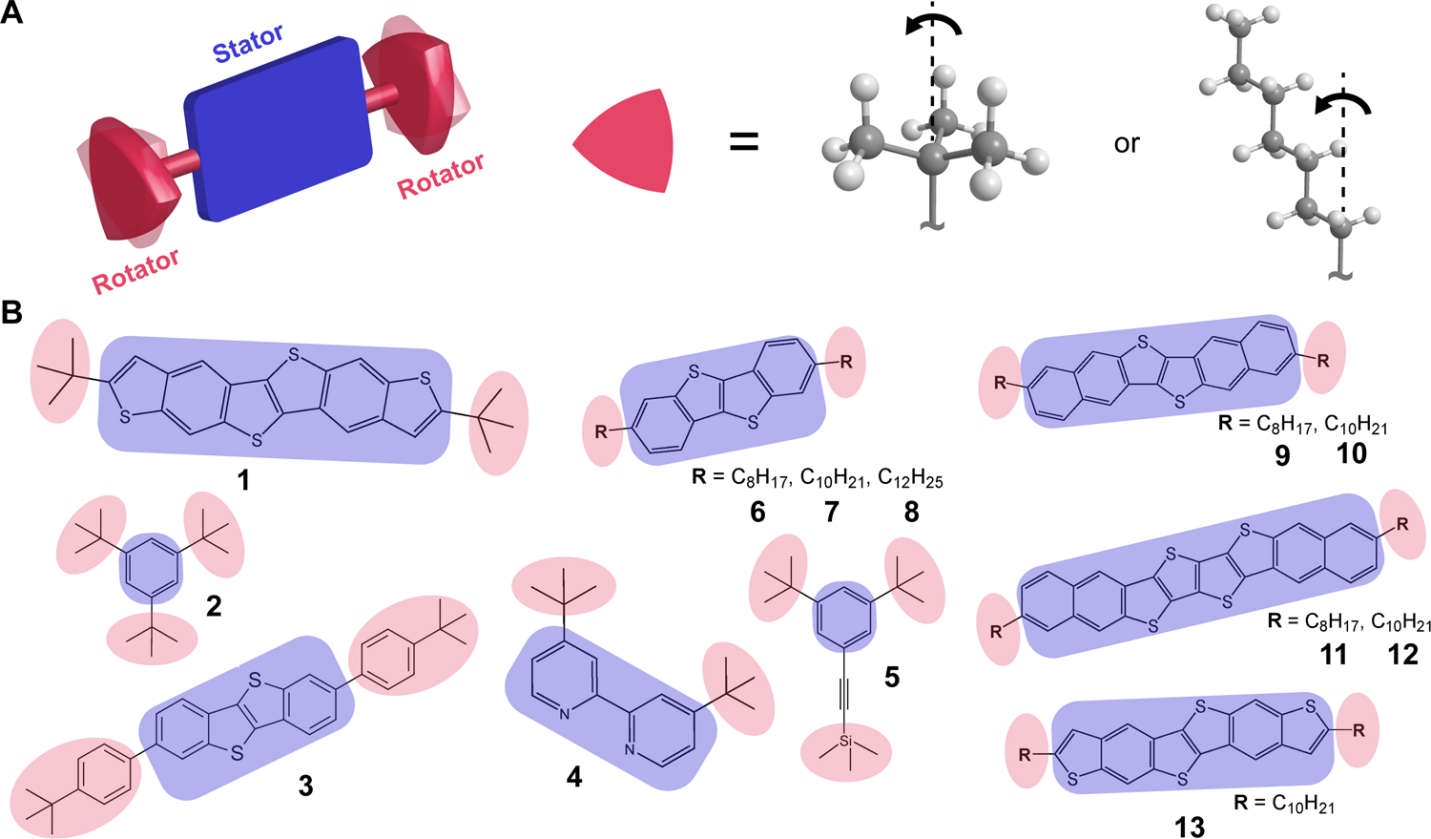
(A) A schematic representation of the molecular strategy exploited to obtain amphidynamic crystals. (B) 13 molecular systems under study with stator and rotator components highlighted in blue and red, respectively.

Generalizing these findings, here, we present a simple and robust strategy to prepare amphidynamic crystals, crystalline materials endowed with long-range ordered structures combined with highly mobile elements^24,25^ (Figure 1). The mobile molecular fragments are designed to undergo a temperature-induced order–disorder transformation that translates molecular-scale dynamics into macroscopic cooperative polymorphic transitions associated with a thermoelastic shapeshifting response. The strategy has been successfully applied to a broad range of different molecules, from simple and commercially available benzene derivatives up to complex high-performance organic semiconductors. This is the first successful attempt to exploit molecular and crystal engineering to access on-demand polymorphism with tailored phase transitions, gaining unprecedented control over organic crystalline materials.

We chose 13 candidates based on a straightforward molecular strategy devised to obtain amphidynamic crystals from molecular building blocks possessing rigid and static aromatic cores (stators), anticipated to have a high moment of inertia, functionalized with highly mobile side chains (rotators) designed to undergo rotational Brownian motion, as shown in Figure 1. We selected molecular systems with different chemical identities, sizes, and shapes to assess the robustness of the proposed model. Crystals of **2**, **4**, **6**, and **10** are known to possess multiple crystal phases and solid-to-solid transitions (see the Supporting Information for further details) as single crystals (**2**, **4**, **6**) and thin films (**10**), but no previous studies have reported the cooperative nature of the phase transitions.^26−30^ Among the stators, there are commercially available (**2**) and custom-synthesized (**5**) benzene derivatives, a 2,2′-bipyridine derivative (**4**), and a series of high-performance organic semiconductive heteroacenes, namely benzothieno[3,2-*b*][1]benzothiophene (**3**, **6**, **7**, **8**), dinaphtho[2,3-*b*:2′,3′-*f*]thieno[3,2-*b*]thiophene (**9**, **10**), thieno[3,2-*f*]thieno[3′,2′:5,6]-[1]benzothieno[3,2-*b*][1]benzothiophene (**1**, **13**), and naphtho[2,3-*b*]thieno-[2‴,3″′:4′′,5′′]thieno[2″,3″:4′,5′]thieno[3′,2′-*b*]naphtho[2,3-*b*]thiophene (**11**, **12**).^31−34^ The rotators were chosen as either bulky alkyl fragments, *tert*-butyl and trimethylsilyl groups, for compounds **1** to **5**, or long linear alkyl chains (C_*n*_ with *n* > 8) for compounds **6** to **13**, given the tendencies of both types of functionalities to undergo temperature-induced dynamic conformational changes.^19,20^

The compresence of an ordered lattice and a Brownian sublattice evolving with temperature has been hypothesized to be a structural condition to likely obtain ordered–disorder cooperative polymorphic transitions driven by specific lattice dynamics.^18^ To test this hypothesis, we crystallized all molecules and characterized selected crystals with differential scanning calorimetry (DSC), heating/cooling stage coupled to polarized optical microscopy (POM), single-crystal X-ray diffraction (SCXRD), low-frequency Raman spectroscopy, a computational approach that includes the calculation of lattice energy and intermolecular interactions based on density functional theory (DFT), and molecular dynamics (MD) simulations to obtain mechanistic insights into the polymorphic transitions and the underpinning criteria for their observation.

## Results and Discussion

**Figure 2:**
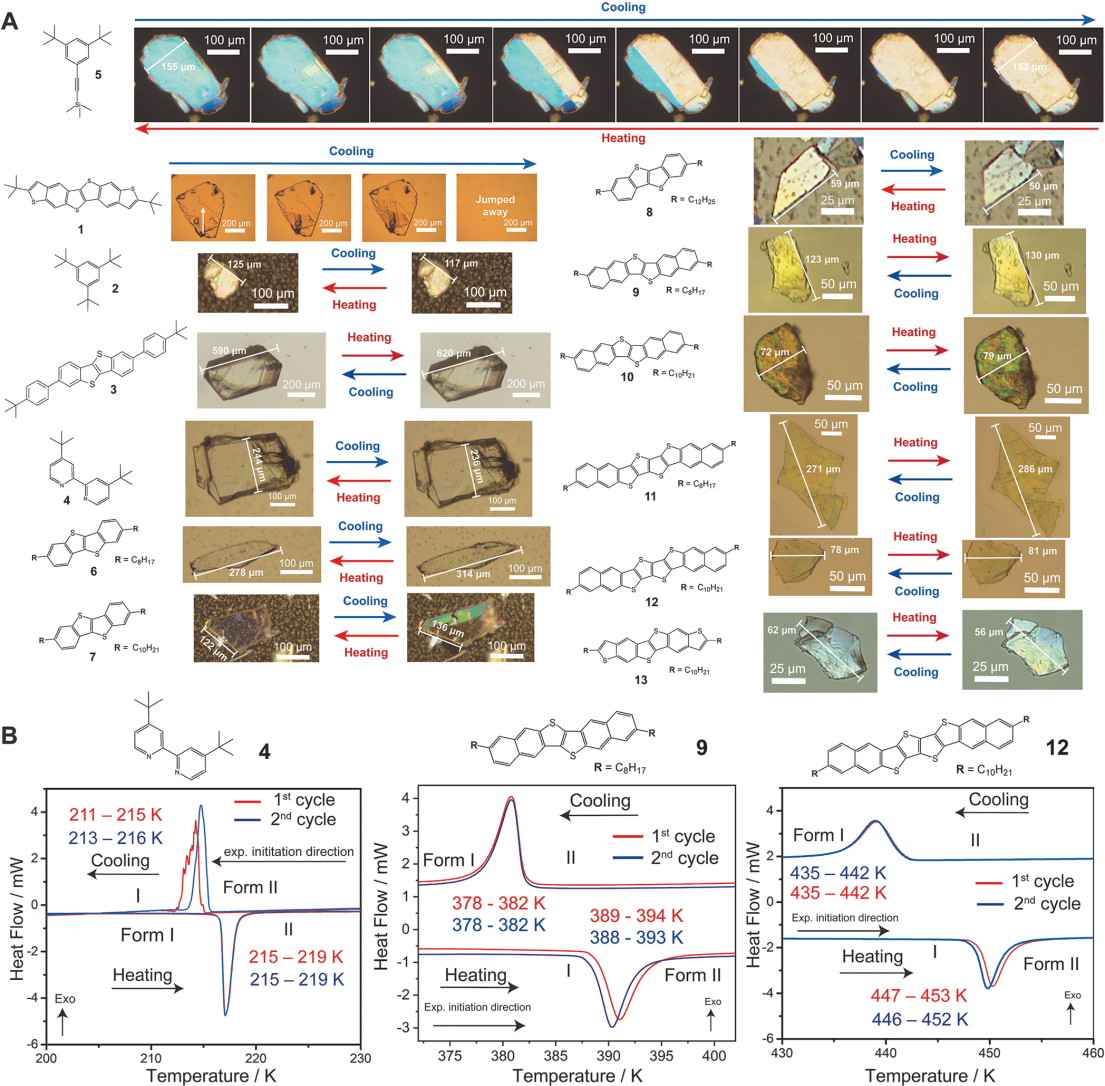
(A) POM images of the cooperative phase transitions of **1**–**13** associated with shapeshifting and the corresponding change in dimensions, clear polymorphic phase front propagation (see **5**), and thermosalience (see **1**). White arrows for the crystal of **1** indicate the phase propagation front direction. POM investigation started at room temperature for all systems followed by cooling or heating depending on the transition temperature of each compound. For each system, the experiment initiation direction is from left to right. (B) DSC plots of selected compounds **4**, **9**, and **12**.

The screening of the crystals by heating/cooling stage with POM in a broad range of temperatures readily revealed the typical signatures of cooperative transitions for all the samples, as shown in Figure 2A (Supporting Information, Movies S1–S17). Specifically, a fast and sharp polymorphic phase front propagation through the solids as distinct from the classic nucleation and growth transition mechanism (e.g., crystals of **5** in Figure 2A, Movie S6), crystals jumping upon phase transitions, namely thermosalience (crystals of **1**, Movies S1 and S2), temperature-induced shapeshifting with the length of the crystals sharply changing at the transition up to 15% (**8**), and reversible transformations for all compounds were observed. The enantiotropic nature of the polymorphic transitions was confirmed by DSC for all samples with phase transformation temperatures as low as 109 K in cooling (sample **6**) and as high as 520 K in heating (sample **3**). The thermal hysteresis gaps varied greatly, from the narrow 2 K hysteresis window observed with **4** to the 179 K hysteresis of the phase transition of **3** (Figure S3), the latter being the largest hysteresis of organic crystal polymorphism reported to date to the best of our knowledge (see Figure 2B, Table S1, and Figures S2–5). Phase transition peaks often showed a sawtooth profile (Figures 2B and S2–S5), this being another typical signature of cooperative transitions in organic crystals. While a general trend of the transition temperatures and thermal behaviors within the series is not evident due to the non-negligible chemical differences of the starting building blocks and the statistically low number of samples, an interesting trend emerges in the homologous series **6**–**8** with the transition temperatures increasing with the increasing length of the side alkyl chains from 8 to 12 carbons as expected due to the increase of the cohesive dispersive interactions with longer alkyl chains (Figure S1).^35^

**Figure 3:**
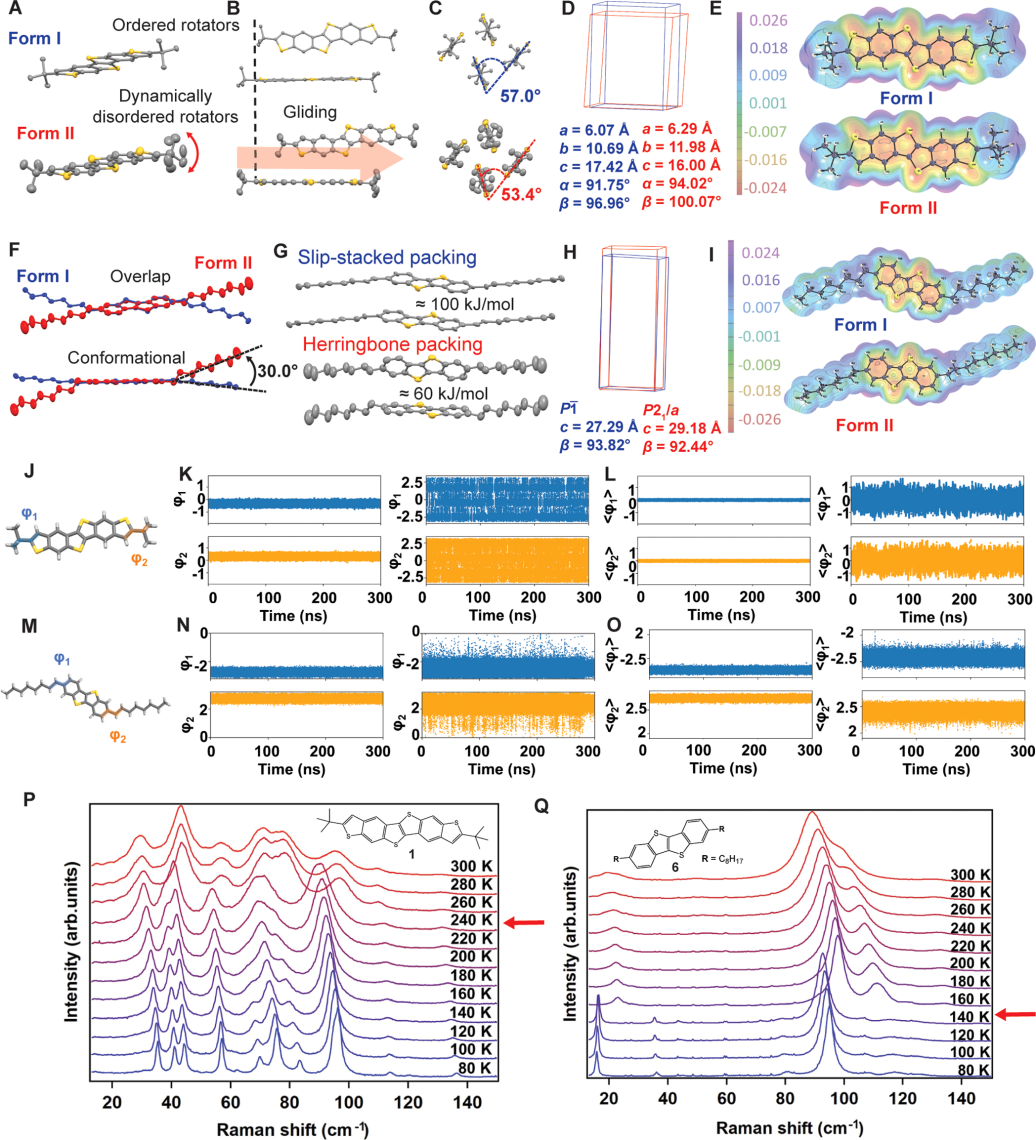
(A) Ordered and disordered side chains of form I (top) and form II (bottom) of **1**. (B) Gliding of the aromatic cores from form I (top) to form II (bottom) of **1**. (C) Changing of the packing angles of the aromatic cores of **1** from form I (top) to form II (bottom). (D) Overlapping unit cells of form I (blue) and form II (red) of **1**. (E) Molecular electrostatic potential (B3LYP aug-pcseg2, resolution 0.04 Å) on the 0.001 au contour of the electron density surface of both polymorphs of **1**. (F) Conformational changes between form I (blue) and form II (red) of **6**. (G) Change in aromatic packing between form I (top) and form II (bottom) of **6**. (H) Overlapping unit cells of form I (blue) and form II (red) of **6**. (I) Molecular electrostatic potential (B3LYP aug-pcseg2, resolution 0.04 Å) on the 0.001 au contour of the electron density surface of both polymorphs of **6**. (J) φ_1_ and φ_2_ angles for **1**. (K) φ_1_ and φ_2_ fluctuations at 100 (left) and 255 K (right) of a single molecule in **1**. (L) <φ_1_> and <φ_2_> fluctuations at 100 K (left) and 255 K (right) of the crystal of **1**. (M) φ_1_ and φ_2_ angles for **6**. (N) φ_1_ and φ_2_ fluctuations at 100 K (left) and 300 K (right) of a single molecule in **6**. (O) <φ_1_> and <φ_2_> fluctuations at 100 K (left) and 300 K (right) of the crystal of **6**. (P and Q) Low-frequency temperature-dependent Raman spectra of **1** (P) and **6** (Q). Red arrows indicate the transition temperature.

To shed light on the cooperative transition mechanisms and to understand the role of the different side chains, we selected compounds **1** and **6** bearing a bulky *tert*-butyl substituent and an octyl side chain, respectively, for an in-depth comparative structural and spectroscopic analysis by combining SCXRD data, DFT and MD simulations, and low-frequency Raman spectroscopy, as shown in Figure 3. The crystal structure of compound **1** at 255 K exhibits a triclinic *P*1̅ space group (form II) with the extended aromatic cores forming the classic herringbone packing generally observed for heteroacene-based crystalline organic semiconductors, Figure 3 and Table S2. The *tert*-butyl substituents show temperature-activated dynamic disorder with the methyl groups refined over two different positions and with large atomic displacement parameters (ADPs), as shown in Figure 3A. If cooled to 100 K, crystals of **1** undergo a thermosalient cooperative transition in the 230–240 K range with the plate-like crystals leaping (Figure 2 and Movies S1 and S2) at the transition temperature. In line with previously reported systems,^19^ the low-temperature polymorph of **1** (form I) retains the same overall symmetry and space group but with significant structural changes, namely the ordering of the *tert*-butyl substituents (Figure 3A), molecular gliding (Figure 3B), a prominent change of the angle of the aromatic cores within the herringbone packing (Figure 3C), and changes in the unit cell parameters that cannot be attributed to thermal expansion, such as the 8% expansion of the *c* axis, the 12% compression of the *b* axis, and the non-negligible variations of the unit cell angles (Figure 3D). Both low-temperature (form I) and high-temperature (form II) polymorphs were previously reported for compound **6.**(30,35) At room temperature, form II shows again the typical herringbone packing of heteroacene aromatic cores as crystals of **1** with disordered side alkyl chains and large ADPs, Figure 3F. If cooled to 100 K, crystals undergo a cooperative transition in a broad temperature range (109–149 K), with a large hysteresis of 81 K and with fragmentation and/or reversible shapeshifting, resulting in up to 12% elongation (Figure 2A and Movies S7–S9). Differently from **1**, the cooperative transition from form II to form I of **6** involves major packing rearrangements, as shown in Figure 3F,G. In particular, the octyl linear side chains undergo a major conformational change (Figure 3F), and the aromatic cores transition from the herringbone packing in form I to a slipped-stacked packing in form II (Figure 3G). The possibility of tuning the aromatic packing of organic semiconductors through a polymorphic transition while preserving single-crystal integrity is remarkable. Tuning of the crystal structure in such a dramatic way is generally achieved by time-demanding, uncertain, and costly molecular or crystal engineering strategies,^36^ and it is expected to have a significant impact on the overall electronic properties of the final functional material.^37^ As a typical order–disorder phase transition, the crystal symmetry of **6** lowers with cooling from the monoclinic *P*2_1_/*c* space group to the triclinic *P*1̅ with minimal changes in the unit cell parameters, mainly the *c* axis and β angle (Figure 3H).

To quantify and compare the intermolecular forces governing the packing of both **1** and **6**, we performed Hirshfeld surface analysis and DFT calculations. We confirmed that both systems are held together by weak dispersive interactions, either slipped-stacked π interactions or edge-to-face π interactions and van der Waals forces between the aliphatic side chains (as detailed in the Supporting Information, Tables S4–S7 and Figures S6–S13). The pair of polymorphs has very similar molecular electrostatic potential surfaces (Figure 3E,I), suggesting dispersion-driven interaction patterns for both compounds as a key and common structural feature. As anticipated from the single SCXRD analysis, the cooperative transition of **6** brings a surprisingly reversible change in the dominant aromatic interactions from the robust slipped-stacked packing (ca. 110 kJ/mol) in form I to the weaker herringbone packing with predominant edge-to-face interactions (*ca*. 60 kJ/mol) in form II.

**Figure 4:**
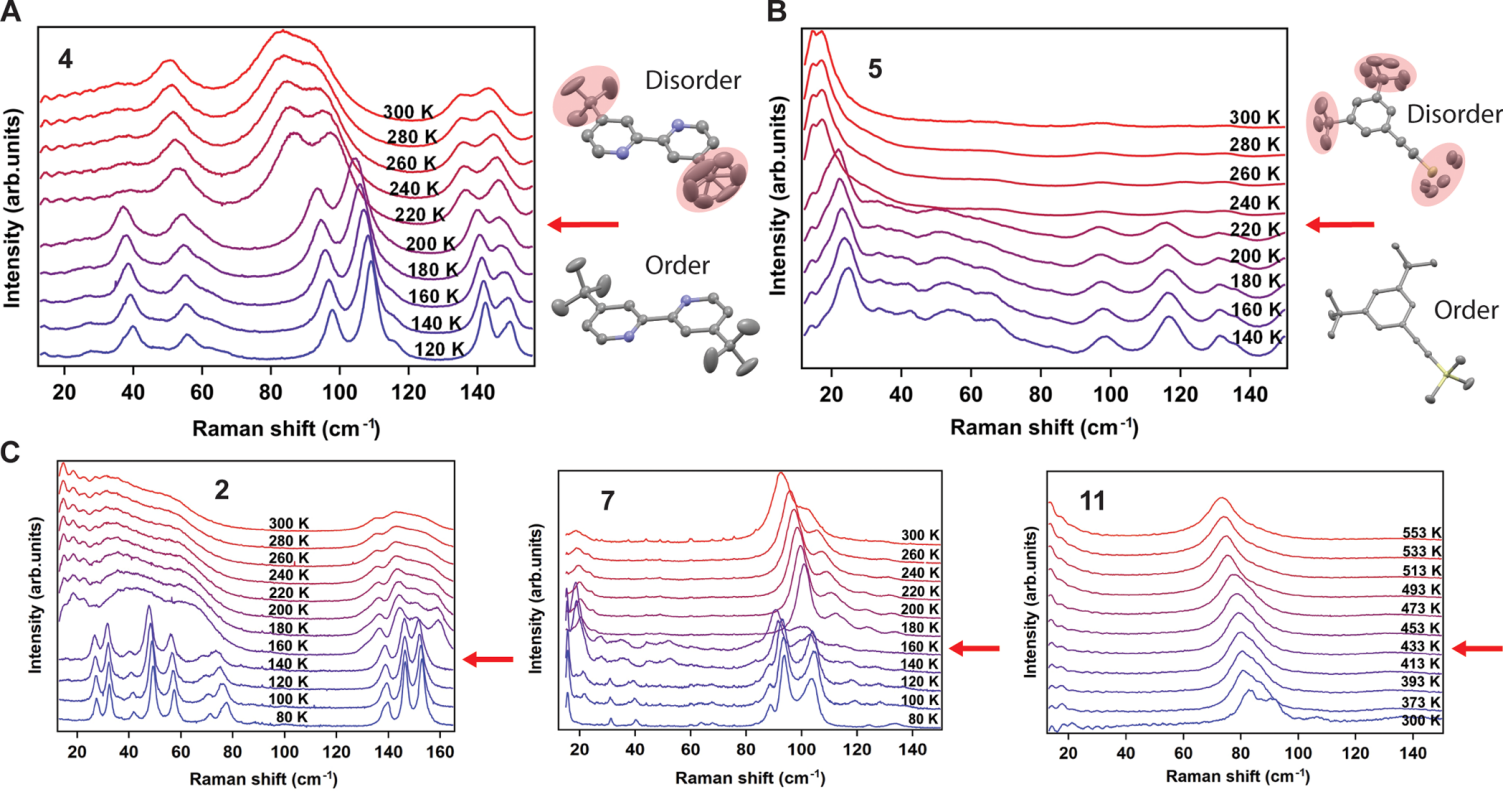
(A) Low-frequency temperature-dependent Raman spectra of **4** (left) and ordered and disordered side chains of the low-temperature form (bottom right) and high-temperature form (top right) of **4** from SCXRD data. (B) Low-frequency temperature-dependent Raman spectra of **5** (left) and ordered and disordered side chains of the low-temperature form (bottom right) and high-temperature form (top right) of **5** from SCXRD data. (C) Low-frequency temperature-dependent Raman spectra of **2** (left), **7** (center), and **11** (right). Red arrows indicate the transition temperature.

We then conducted classical MD simulations on **1** and **6** to gain molecular and mechanistic insights into the polymorphic transitions. The simulations maintained the crystalline matrix at 255 and 100 K for **1** and at 300 and 100 K for **6**. We observed the dynamics of the generated supercells to explore the conformational behavior of these systems below and above the transition temperature. We obtained similar results using 2 × 2 × 2 (Figure 3J–O) or 4 × 4 × 4 supercells (Figure S18). We monitored each side chain dihedral angle, defined as φ_1_ and φ_2_ for **1** and **6**, respectively (Figure 3J,M). We observed the change in dihedral angles for a single representative molecule in the cell (Figure 3 K,N). In addition, we calculated the ensemble average over all the molecules in the supercell (<φ_i_>) as in Figure 3L, O. For **1** at 100 K, the average dihedral angles fluctuate around their initial value (<φ_i_ ≥ 0, Figure 3L), while at 255 K, the average dihedral angles show rapid rotation transitions (Figure 3L). At higher temperatures, the dihedral angles mainly rotate around three values (Figure 3L). The average, counting for all of the molecules in the cell, shows fluctuations. However, the average includes the cancelation of opposite transitions. Similarly, for **6** at 100 K, <φ_1_> and <φ_2_> fluctuate around their initial values. At 300 K, there are vigorous fluctuations and transitions between conformations, and the ensemble trajectory-based average side chain dihedral angles (<φ_1_> and <φ_2_>) are shifted 20–30° relative to the values at 100 K (Figure 3O). To further explore side chain dynamics below and above the transition temperature in **1** and **6**, the distribution of the observed value during the MD trajectory was evaluated by computing a reaction coordinate that reflects the dynamic transition for each material at low and high temperatures (for more information, see the Supporting Information). For **1** at 100 K, a single basin is observed (Figure S18C inset). However, at 255 K, a broader sampling range with additional conformations is observed (Figure S18C). A similar trend is obtained in the case of **6**, where at the lower temperature (100 K), we observed a single basin (Figure S18F inset), while at 300 K, we see a wider sampling range (Figure S18F). In both systems, MD simulations confirmed a temperature-induced dynamic behavior of the side chains consistent with the expected order–disorder phase transitions. This establishes a dynamic behavior similar to previously reported amphidynamic crystals undergoing cooperative phase transitions,^18^ validating our initial molecular and structural design concept.

To experimentally characterize the order–disorder nature of the observed cooperative transitions and to further understand the link between polymorphism and molecular and lattice dynamics beyond the averaged and static depiction obtained from XRD data, we performed temperature-dependent low-frequency (<200 cm^–1^) Raman spectroscopy measurements. This technique is ideally suited for studying crystalline structural transformations,^38^ as preliminary experiments have shown for **6** and other cooperative transitions.^17,18,30^Figure 3P, Q shows the temperature-dependent Raman scattering from 80 to 300 K for single crystals of **1** and **6**, respectively. Passing from form I to form II for both **1** and **6** results in an abrupt blue shift and broadening of the spectra between 140 and 160 K for **6** and between 240 and 260 K for **1** (see also Figure S14). These results align well with the temperature range of the cooperative transitions observed by thermal and XRD analyses. The abrupt changes of the vibrational features across the phase transitions, such as peak-shifting and broadening, are consistent with an order–disorder process suddenly increasing the rotational dynamics of the side chains of both systems and with significant anharmonic behavior of the low-frequency vibrations,^18^ further corroborating the success of our strategy to obtain polymorphic crystalline materials possessing specific molecular and lattice dynamics.

In accordance with the observations for **1** and **6**, similar temperature-induced order–disorder dynamics of the rotator side chains and slight structural rearrangements were observed for the reported crystals of **2** and **4**(27,28) and in crystals of **5** via SCXRD (Figures 4A,B and S17, and Table S3). To further confirm the generality of our findings and the prominent molecular-scale dynamics involved in the polymorphic transitions, we performed additional low-frequency Raman spectroscopy experiments on selected compounds (**2**, **4**, **5**, **7**, and **11**), as shown in Figures 4 and S15–S16. All systems displayed sudden shifting and broadening of the normal modes of vibration below 200 cm^–1^ across the phase transitions as observed for the cooperative phase transitions of crystals of **1** and **6**.

## Conclusions

We have developed a simple molecular strategy that combines static aromatic cores functionalized with dynamic alkyl side chains (bulky functional groups or long linear alkyl chains) in a series of 13 amphidynamic crystals. The molecular building blocks are engineered to selectively undergo order–disorder phase transitions. All the crystalline systems show temperature-induced cooperative phase transitions associated with specific molecular and lattice dynamics that result in macroscopic effects, namely, thermosalience and crystal shapeshifting, paving the way for a rational design of crystal polymorphism and crystalline functional materials with switchable properties, expanding the scope of crystal engineering beyond the classic static representation of crystal structures and their molecular building blocks. The present strategy is readily generalizable, and future studies focused on expanding the series of cooperative transitions to new classes of crystalline materials would be of great value to further shed light on the complex dynamics–structure–property relationship in organic crystal chemistry. In this context, developing crystal structure prediction methods that take into account the inherent dynamic nature of organic crystals would assist the experimental search for novel dynamic crystalline materials.

## Supporting Information Available

Materials, synthetic procedures, crystal growth, thermal, spectroscopic, and structural characterization, optical imaging, DFT, and MD (PDF)
Thermosalient effect of crystal of **1**: sample 1 (ZIP)
Thermosalient effect of crystal of **1**: sample 2 (ZIP)
Cooperative transition and shapeshifting of the crystal of **2** (ZIP)
Cooperative transition and shapeshifting of the crystal of **3** (ZIP)
Cooperative transition and shapeshifting of the crystal of **4** (ZIP)
Cooperative transition and phase front propagation of the crystal of **5** (ZIP)
Cooperative transition and shapeshifting of the crystal of **6**: sample 1 (ZIP)
Cooperative transition and shapeshifting of the crystal of **6**: sample 2 (ZIP)
Cooperative transition and shapeshifting of the crystal of **6**: sample 3 (ZIP)
Cooperative transition and shapeshifting of the crystal of **7** (ZIP)
Cooperative transition and shapeshifting of the crystal of **8** (ZIP)
Cooperative transition and shapeshifting of the crystal of **9:** sample 1 (ZIP)
Cooperative transition and shapeshifting of the crystal of **9**: sample 2 (ZIP)
Cooperative transition and shapeshifting of the crystal of **10** (ZIP)
Cooperative transition and shapeshifting of the crystal of **11** (ZIP)
Cooperative transition and shapeshifting of the crystal of **12** (ZIP)
Cooperative transition and shapeshifting of the crystal of **13** (ZIP)
