# Supplementary material for: Toward On-Demand Polymorphic Transitions of Organic Crystals via Side Chain and Lattice Dynamics Engineering
Source: J Am Chem Soc. 2024 Nov 8;146(46):31911–9. doi: 10.1021/jacs.4c11289 (PMC11583316; doi:10.1021/jacs.4c11289)
Supplement: Supplementary file 1 — ja4c11289_si_001.pdf [file ja4c11289_si_001.pdf]

Supporting Information

Towards On-Demand Polymorphic Transitions of Organic Crystals *via* Side Chain and Lattice Dynamics Engineering

Luca Catalano[†,ǂ,§,*], Rituraj Sharma[‡,Ɩ], Durga Prasad Karothu[ǁ], Marco Saccone[⊥], Oren Elishav[±], Charles Chen[±], Navkiran Juneja[ǂ], Martina Volpi[†], Rémy Jouclas[†], Hung-Yang Chen[¶], Jie Liu[†,⅂], Guangfeng Liu[†,¬], Elumalai Gopi[†], Christian Ruzié[†], Nicolas Klimis[¤], Alan R. Kennedy[⁅], T. Kyle Vanderlick[±], Iain McCulloch[∇,∏], Michael T. Ruggiero[ǂ], Pančе Naumov[ǁ,∫,∬,∲], Guillaume Schweicher[†], Omer Yaffe[‡], and Yves H. Geerts[†,⊕]

† Laboratoire de Chimie des Polymères, Université Libre de Bruxelles (ULB), Brussels, 1050, Belgium.

ǂ Department of Chemistry, University of Rochester, Rochester, NY 14627, USA.

§ Dynamic Molecular Materials Laboratory, Dipartimento di Scienze della Vita, Università degli Studi di Modena e Reggio Emilia, Modena, 41125, Italy.

‡ Department of Chemical and Biological Physics, Weizmann Institute of Science, Rehovot, 76100, Israel.

Ɩ Centre for Scientific and Applied Research (CSAR), IPS Academy, Indore, 452012, India.

ǁ Smart Materials Lab, New York University Abu Dhabi, Abu Dhabi, 129188, UAE.

⊥ Dipartimento di Scienze e Innovazione Tecnologica, Università del Piemonte Orientale, Alessandria, 15121, Italy.

± Department of Chemical and Environmental Engineering, Yale University, New Haven, CT 06520, USA.

¶ Department of Chemistry and Centre for Plastic Electronics, Imperial College London, London, SW7 2AZ, UK.

⅂ Department of Physics, University of Warwick, Coventry CV47AL, U.K.

¬ Jiangsu Key Laboratory of Advanced Catalytic Materials & Technology, School of Petrochemical Engineering, Changzhou University, Changzhou, 213164, P.R. China.

¤ Ohme; Brussels, 1050, Belgium.

⁅ Department of Pure and Applied Chemistry, University of Strathclyde, Glasgow, G1 1XL, UK.

∇ Andlinger Center for Energy and the Environment, and the Department of Electrical and Computer Engineering, Princeton University, Princeton, NJ 08544, USA.

∏ Department of Chemistry, Chemistry Research Laboratory, University of Oxford, Oxford, OX1 3TA, UK.

∫ Center for Smart Engineering Materials, New York University Abu Dhabi, Abu Dhabi, 129188, UAE.

∬ Research Center for Environment and Materials, Macedonian Academy of Sciences and Arts, Skopje, MK-1000, Macedonia.

∲ Molecular Design Institute, Department of Chemistry, New York University, New York, NY 10003, USA.

⊕ International Solvay Institutes of Physics and Chemistry, Brussels, 1050, Belgium.

Corresponding author: luca.catalano@unimore.it

Table of Contents

Materials **3**
Synthetic procedures **3**
Synthesis of **1** 3
Synthesis of **11** & **12** 5
Synthesis of **13** 9
Crystal Growth **10**
Polarized Optical Microscopy **10**
Differential Scanning Calorimetry **10**
Single crystal X-ray diffraction **11**
Raman Spectroscopy **11**
DFT and Hirshfeld surface analysis **12**
Molecular Dynamics **12**
Tables and Figures **14**
Supporting references **40**

Materials

All reagents were purchased Sigma-Aldrich (now Merck), VWR, Strem Chemicals, Acros, Alfa Aesar, abcr, TCI and Fluorochem and were used without further purification. Technical grade solvents were purchased from Chem-Lab and used as supplied. Anhydrous solvents as chloroform, dichloromethane, N,N-dimethylformamide and tetrahydrofurane were distilled using common methods. Air- and/or moisture-sensitive liquids and solutions were transferred via a syringe or a Teflon cannula. Analytical thin-layer chromatography (TLC) was performed on aluminum plates with 10-12 µm silica gel containing a fluorescent indicator (Merck silica gel 60 F254). TLC plates were visualized by exposure to ultraviolet light (254 nm and 365 nm). Flash column chromatography was performed on Grace Davisil LC60A (70-200µm) silica. GC-MS were collected on a Shimadzu GCMS-QP2010. All NMR spectra were recorded on Jeol 400 MHz spectrometer or on a Bruker AV-400 spectrometer at 298 K and are reported in ppm relative to TMS. IR spectra were collected on a Perkin Elmer Spectrum 3 FTIR at room temperature with an ATR setup. Compounds **2** and **4** were purchased from Sigma Aldrich (now Merck), and they were used without further manipulation. All reactions were carried out under an inert Argon atmosphere unless otherwise stated. **3**, **5-8** were synthesized following reported procedures and used without further manipulation for crystallization experiments[35,39-40]. Compounds **9** and **10** were supplied by Nippon Kayaku. Synthesis of **1**, **11**, **12**, and **13** are provided in the supplementary information.

Synthetic procedures

Synthesis of **1**

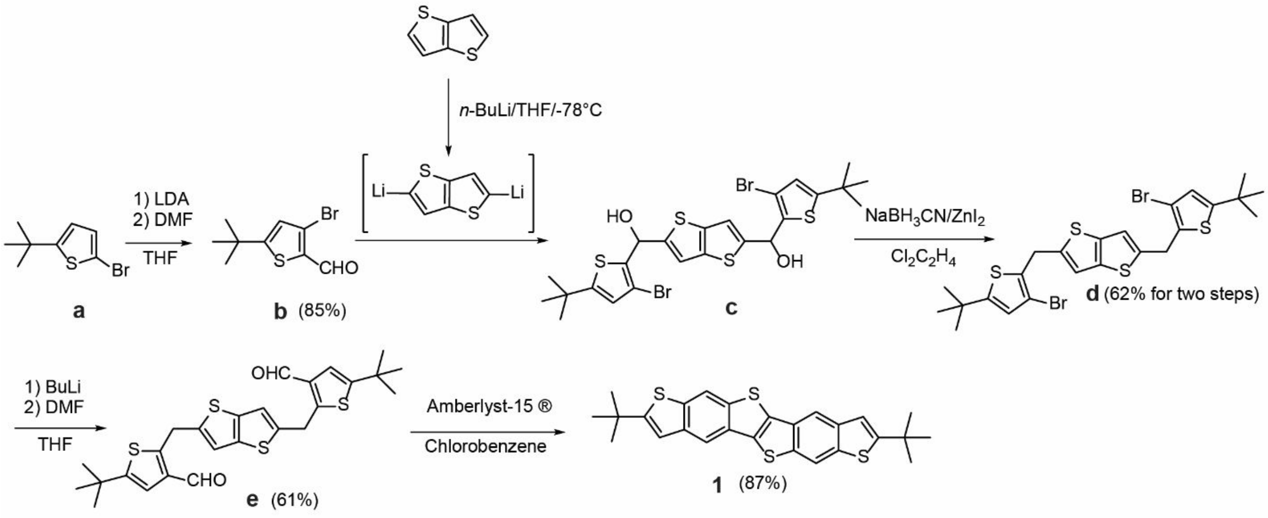

Scheme S1. Synthetic route of **1**.

3-bromo-5-(*tert*-butyl)thiophene-2-carbaldehyde (b): Under argon a solution of LDA 2M in THF/n-heptane/ethylbenzene (10.40 mL, 20.80 mmol) was added dropwise to a solution of compound **a** (3.63 g, 16.57 mmol) in anhydrous THF (50 mL) cooled to -78°C. After stirring for 2h at -78 °C, DMF (5.00 mL, 64.57 mmol) was added and the reaction mixture was slowly warmed to room temperature, the stirring was maintained for 1 h. After

dilution with diethyl ether (100 mL), the mixture was washed with a saturated aqueous solution of NH_4Cl (100 mL), dried over $MgSO_4$, filtered and concentrated to dryness. The resulting oil was purified by chromatography on silica gel (eluent: hexane/DCM: 4/1), a yellow oil was obtained (3.49 g, 85% yield). ^1H NMR (300 MHz, $CDCl_3$) δ: 9.89 (s, 1H), 6.91 (s, 1H), 1.39 (s, 9H). ^{13}C NMR (75 MHz, $CDCl_3$) δ: 183.0, 168.2, 134.1, 127.2, 120.2, 35.7, 31.9. EI-HRMS ($C_9H_{11}BrOS$): [$M^{•+}$]: calcd mass: 245.9714. found: 245.9711.

2,5-bis((3-bromo-5-(*tert*-butyl)thiophen-2-yl)methyl)thieno[3,2-*b*]thiophene (d): A solution of *n*-BuLi 2.5 M in hexane (6.25 mL, 15.63 mmol) was added slowly to a solution of thieno[3,2-*b*]thiophene (1.00 g, 7.13 mmol) in anhydrous THF (25 mL) cooled to -78 °C under argon atmosphere. After 5 min of stirring at -78 °C, the reaction mixture was slowly warmed to room temperature and stirring during 1 h. Thereafter the reaction mixture was cooled to reach -78 °C, and compound **b** (3.52 g, 14.26 mmol) was added dropwise. The reaction mixture was slowly warmed to room temperature and stirring during 12 h. After addition of diethyl ether (100 mL), the mixture was washed with a saturated aqueous solution of NH_4Cl (100 mL), dried over $MgSO_4$, filtered and concentrated to dryness. The product **c** was directly engaged in the next step without purification. Diol compound was dissolved in 1,2-dichloroethane (175 mL), $NaBH_3CN$ (6.75 g, 107.48 mmol) and ZnI_2 (6.85 g, 21.46 mmol) were added. The reaction mixture was stirred for 20 h at room temperature and then filtered on a celite pad. The solvent was evaporated and the resulting solid was dissolved in DCM (150 mL) before addition of water (100 mL). The organic layer was dried over $MgSO_4$, filtered and the solvent was evaporated. After chromatography on silica gel (eluent: hexane) a white solid was obtained (2.68 g, 62% yield). ^1H NMR (300 MHz, $CDCl_3$) δ: 6.99 (s, 2H); 6.66 (s, 2H), 4.27 (s, 4H), 1.32 (s, 18H). ^{13}C NMR (75 MHz, $CDCl_3$) δ: 156.1, 143.1, 137.7, 134.0, 124.3, 118.0, 108.1, 34.8, 32.2, 30.8. EI-HRMS ($C_{24}H_{26}Br_2S_4$): [$M^{•+}$]: calcd mass: 599.9284, found: 599.9294.

2,2′-(thieno[3,2-*b*]thiophene-2,5-diylbis(methylene))bis(5-(*tert*-butyl)thiophene-3-carbaldehyde) (e): Under argon atmosphere at -78 °C, to a solution of *n*-BuLi 2.5 M in hexane (3.12 mL, 7.81 mmol) in anhydrous THF (30 mL), was added dropwise a solution of compound **d** (1.12 g, 1.86 mmol) in anhydrous THF (20 mL). After 20 min of stirring at -78 °C, DMF (2.0 mL, 25.83 mmol) was added dropwise. After 1 h of additional stirring at this temperature the solution was quenched by addition of water (100 mL). After which the aqueous layer was extracted with diethyl ether (100 mL). The organic layer was dried over $MgSO_4$, filtered, and concentrated to dryness. The crude product was triturated in cold hexane and filtered to give a white powder (570 mg, 61% yield). ^1H NMR (300 MHz, $CDCl_3$) δ: 10.01 (s, 2H), 7.10 (s, 2H), 7.00 (s, 2H), 4.68 (s, 4H), 1.34 (s, 18H). ^{13}C NMR (75 MHz, $CDCl_3$) δ: 184.8, 156.2, 151.4, 143.2, 137.9, 136.4, 121.5, 118.3, 34.7, 32.3, 29.4. EI-HRMS ($C_{26}H_{28}O_2S_4$): [$M^{•+}$]: calcd mass: 500.0972, found: 500.0978.

dibenzo bis(5-*tert*-butylthiopheno)[6,5-*b*:6′,5′-*f*]thieno[3,2-*b*]thiophene (1): To a solution of compound **e** (470 mg, 0.94 mmol) in chlorobenzene (50 mL) was added Amberlyst-15® (2.00 g) and the reaction was refluxed for 12 h under argon atmosphere. The solution was filtered to remove the resin and the solvent evaporated. After recrystallization from toluene, a white solid was obtained (380 mg, 87% yield). ^1H NMR (300 MHz, $CDCl_3$) δ: 8.27 (s, 2H), 8.12 (s, 2H), 7.13 (s, 2H), 1.49 (s, 18H). ^{13}C NMR (75

MHz, $CDCl_3$) δ: 158.8, 138.5, 138.5, 137.2, 132.5, 131.1, 117.5, 117.0, 115.0, 35.3, 32.2. UV/Vis (DCM), λ_{max} (nm): 256, 278, 289, 299, 339, 350, 361. EI-HRMS ($C_{26}H_{24}S_4$): [$M^{\bullet+}$]: calcd mass: 464.0761, found: 464.0753.

Synthesis of **11** & **12**

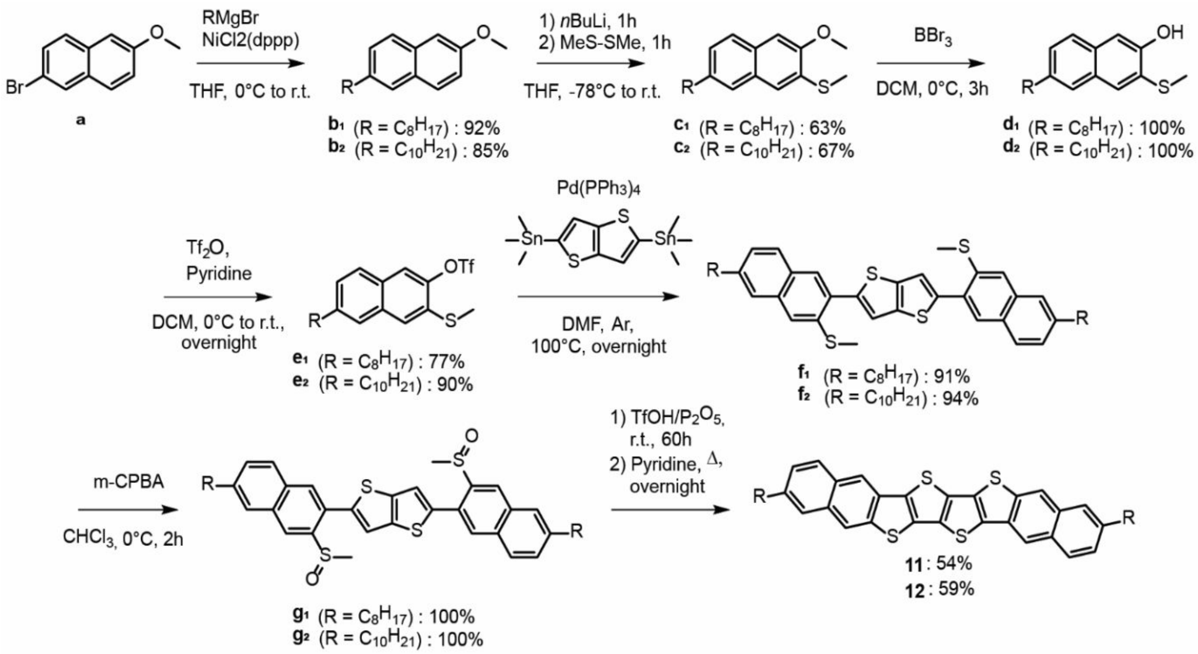

Scheme S2. Synthetic route of **11** and **12**

Experimental procedures

General: THF and DMF were distilled by typical methods, all reactions were performed under argon atmosphere except otherwise mentioned. Dimethyldisulfide was dried over molecular sieves for 1 night, $Pd(PPh_3)_4$ was washed with methanol then diethylether before use.

6-octyl-2-methoxynaphthalene (b_1)[41] **:** To a solution of 6-bromo-2-methoxynaphthalene (4.74 g, 20.0 mmol, 1 eq.), $NiCl_2$(dppp) (271 mg, 500 µmol, 2.5 mol%) in dry THF (20 mL) at 0°C was added octylmagnesium bromide (1M/Et_2O, 24.0 mL, 24.0 mmol, 1.2 eq.) dropwise within 40 min, then the ice bath was removed and the mixture was stirred at room temperature overnight. The reaction was quenched with water, the volatiles were removed under reduced pressure and the resulting aqueous layer was extracted 3 times by EtOAc. The combined organic layers were dried over $MgSO_4$, filtered, and evaporated to give 6.57 g of crude product that was purified by column chromatography (Peth. Ether/DCM 95:5 to 90:10) to afford 4.99 g of pure $\mathbf{b_1}$ with 92% yield. ^1H NMR (400 MHz, $CDCl_3$, δ): 7.67 (d, J = 9.6 Hz, 1H), 7.66 (d, J = 8.4 Hz, 1H), 7.53 (s, 1H), 7.29 (dd, J = 8.4, 1.8 Hz, 1H), 7.13-7.10 (m, 2H), 3.91 (s, 3H), 2.73 (t, J = 7.8 Hz, 2H), 1.72-1.64 (m, 2H), 1.40-1.20 (m, 10H), 0.88 (t, J = 7.0 Hz, 2H).

6-decyl-2-methoxynaphthalene (b_2)[42] **:** To a solution of 6-bromo-2-methoxynaphthalene (8.0 g, 33.7 mmol, 1 eq.), $NiCl_2$(dppp) (366 mg, 675 µmol, 2 mol%) in dry THF (250 mL) at 0°C was added decylmagnesium bromide (1M/Et_2O, 40.5 mL, 1.2 eq.) dropwise within 40 min, then the ice bath was removed, and the mixture was stirred at room temperature overnight. The reaction was quenched with water, the volatiles were removed under reduced pressure and the resulting aqueous layer was extracted 3 times by EtOAc. The combined organic layers were dried over $MgSO_4$, filtered and evaporated to give 11.4 g of crude product that was purified by column chromatography (Heptane/EtOAc 99:1) to afford 8.50 g of pure $\mathbf{b_2}$ with 85% yield. ^{1}H NMR (400 MHz, $CDCl_3$, δ): 7.67 (d, J = 9.7 Hz, 1H), 7.66 (d, J = 8.3 Hz, 1H), 7.53 (s, 1H), 7.29 (dd, J = 8.5, 1.8 Hz, 1H), 7.14-7.09 (m, 1H), 7.10 (s, 1H), 3.91, (s, 3H), 2.73 (t, J = 7.4 Hz, 2H), 1.68 (m, 2H), 1.41-1.20 (m, 14H), 0.88 (t, J = 7.0 Hz, 3H).

Typical procedure for the 3-methylthiolation via selective lithiation on 2-methoxynaphthalenes

6-octyl-2-methoxy-3-methylthionaphthalene (c_1): To a solution of $\mathbf{b_1}$ (4.99 g, 18.5 mmol, 1 eq.) in dry THF (18.5 mL) at 0°C was added *n*BuLi (2.5 M/Hexanes, 8.25 mL, 19.4 mmol, 1.05 eq) dropwise, and the mixture was stirred at room temperature for 1h. Then the mixture was cooled again to 0°C and dry dimethyl disulfide (2.0 mL, 22.15 mmol, 1.2 eq.) was added dropwise, and the mixture was stirred at room temperature overnight. The volatiles were removed under reduced pressure, the residue was dissolved in a mixture of aqueous NH_4Cl (10% w/w, 200 mL) and EtOAc (200 mL), the phases were separated, and the aqueous layer was extracted with EtOAc (200 mL). The combined organic layers were dried over $MgSO_4$, filtered and evaporated to give 6.7 g of crude product that was repeatedly recrystallized in EtOH to afford 3.69 g of pure $\mathbf{c_1}$ with 63% isolated yield. ^{1}H NMR (400 MHz, $CDCl_3$, δ): 7.62 (d, *J* = 8.4 Hz, 1H), 7.48 (s, 1H), 7.41 (s, 1H), 7.23 (dd, *J* = 8.2, 1.7 Hz, 1H), 7.05 (s, 1H), 3.99 (s, 3H), 2.72 (t, *J* = 7.5 Hz, 2H), 2.53 (s, 3H), 1.72-1.64 (m, 2H), 1.40-1.20 (m, 10H), 0.88 (t, *J* = 6.7 Hz, 3H). ^{13}C NMR (101 MHz, $CDCl_3$, δ): 154.19, 138.85, 130.57, 129.57, 129.53, 127.13, 126.43, 125.14, 123.03, 104.79, 55.99, 36.10, 32.03, 31.60, 29.65, 29.48, 29.41, 22.81, 14.72, 14.24. HRMS (EI-GCMS) *m/z*: $[M]^+$ calcd for $C_{20}H_{28}OS$, 316.1861; found, 316.1867. TLC (Heptane/EtOAc 4:1): Rf = 66%.

6-decyl-2-methoxy-3-methylthionaphthalene (c_2)[42] **:** 67% yield from 8.50 g. ^{1}H NMR (400 MHz, $CDCl_3$, δ): 7.62 (d, *J* = 8.3 Hz, 1H), 7.48 (s, 1H), 7.41 (s, 1H), 7.23 (dd, *J* = 8.3, 1.8 Hz, 1H), 7.05 (s, 1H), 3.99 (s, 3H), 2.72 (t, *J* = 7.7 Hz, 2H), 2.53 (s, 3H), 1.72-1.64 (m, 2H), 1.40-1.20 (m, 14H) 0.88 (t, *J* = 6.7 Hz, 3H).

Typical procedure for the selective demethylation on the methoxy group of 2-methoxy-3-methylthionaphthalenes

6-octyl-3-methylthio-naphthol (d_1): To a solution of $\mathbf{c_1}$ (3.69 g, 11.7 mmol, 1 eq.) in dry DCM (55 mL) at 0°C was added BBr_3 (1M/DCM, 23.3 mL, 23.3 mmol, 2.0 eq.) dropwise, and the mixture was stirred at room temperature for 3h. The reaction was quenched by addition of ice, the phases were separated, and the organic layer was extracted with

DCM. The combined organic layers were dried over $MgSO_4$, filtered and evaporated to give 3.52 g of **d_1** with quantitative yield. ^1H NMR (400 MHz, $CDCl_3$, δ): 7.94 (s, 1H), 7.61 (d, *J* = 8.4 Hz, 1H), 7.48 (s, 1H) 7.28 (dd, *J* = 8.4, 1.7 Hz, 1H), 7,28 (s, 1H), 6.55 (s, 1H), 2.71 (t, *J* = 7.7 Hz, 2H), 2.42 (s, 3H), 1.71-1.63 (m, 2H), 1.40-1.20 (m, 10H), 0.88 (t, *J* = 6.7 Hz, 3H). ^{13}C NMR (101 MHz, $CDCl_3$, δ): 152.25, 138.60, 133.69, 133.62, 129.30, 128.87, 126.44, 125.80, 124.24, 109.17, 36.04, 32.03, 31.51, 29.64, 29.47, 29.41, 22.81, 20.02, 14.24. HRMS (EI-GCMS) *m/z*: $[M]^+$ calcd for $C_{19}H_{26}OS$, 302.1704; found, 302.1702. TLC (Heptane/EtOAc 4:1): Rf = 42%.

6-decyl-3-methylthio-naphthol (d_2)[42] : quantitative yield from 8.5 g. ^1H NMR (400 MHz, $CDCl_3$, δ): 7.94 (s, 1H), 7.61 (d, *J* = 8.5 Hz, 1H), 7.48 (s, 1H), 7.28 (dd, *J* = 8.4, 1.7 Hz, 1H), 7.28 (s, 1H), 6.54 (s, 1H), 2.71 (t, *J* = 7.7 Hz, 2H), 2.42 (s, 3H), 1.72-1.62 (m, 2H), 1.40-1.20 (m, 14H), 0.88 (t, *J* = 6.8 Hz, 3H).

Typical procedure for the synthesis of 3-methylthio-2-naphthyl trifluoromethanesulfonates

6-octyl-3-methylthio-2-naphthyl trifluoromethanesulfonate (e_1): To a solution of **d_1** (2.32 g, 7.68 mmol, 1 eq.) and pyridine (1.06 mL, 24.2 mmol, 3.15 eq.) in dry DCM (60 mL) at 0°C was added trifluoromethanesulfonic anhydride (1.49 mL, 8.83 mmol, 1.15 eq.) dropwise, and the mixture was stirred at room temperature overnight. The reaction was quenched by addition of aqueous HCL (1M, 50 mL), the phases were separated and the organic layer was extracted with DCM (50 mL). the combined organic layers were dried with $MgSO_4$, filtered and evaporated to give 3.22 g of crude product that was subjected to column chromatography (Heptane/DCM 95:5) to afford 2.58 g of **e_1** with 77% yield. ^1H NMR (400 MHz, $CDCl_3$, δ): 7.72 (d, *J* = 8.4 Hz, 1H), 7.68 (s, 1H), 7.64 (s, 1H), 7.57 (s, 1H), 7.36 (dd, *J* = 8.4, 1.7 Hz, 1H), 2.76 (t, *J* = 7.7 Hz, 2H), 2.59 (s, 3H), 173-1.65 (m, 2H), 1.40-1.20 (m, 10H), 0.88 (t, *J* = 6.7 Hz, 3H).^{13}C NMR (101 MHz, $CDCl_3$, δ): 145.01, 142.84, 133.12, 130.83, 129.63, 128.54, 127.80, 126.57, 125.38, 119.31, 118.84 (q, *J* = 320.5 Hz), 36.23, 32.01, 31.33, 29.61, 29.42, 29.38, 22.80, 15.96, 14.23.HRMS (EI-GCMS) *m/z*: $[M]^+$ calcd for $C_{20}H_{25}O_3F_3S_2$, 434.1197; found, 434.1207. TLC (Heptane/EtOAc 9:1): Rf = 59%.

6-decyl-3-methylthio-2-naphthyl trifluoromethanesulfonate (e_2)[42]: 90% yield from 8.28 g.^1H NMR (400 MHz, $CDCl_3$, δ): 7.72 (d, *J* = 8.4 Hz, 1H), 7.68 (s, 1H), 7.64 (s, 1H), 7.57 (s, 1H), 7.36 (dd, *J* = 8.4, 1.7 Hz, 1H), 2.76 (t, *J* = 7.7 Hz, 2H), 2.59 (s, 3H), 1.73-1.65 (m, 2H), 1.40-1.20 (m, 14H), 0.88 (t, *J* = 6.8 Hz, 3H).

Typical procedure for the Stille cross-coupling between 2,5-bis-trimethylstannylthieno[3,2-b]thiophene and 3-methylthio-2-naphthyl trifluoromethylsulfonates

2,5-bis-(6-octyl-3-methylthio-2-naphtyl)thieno[3,2-*b*]thiophene (f_1): **e_1** (1.15 g, 2.66 mmol, 2 eq.), 2,5-bis-trimethylstannylthioeno[3,2-*b*]thiophene (619 mg, 1.33 mmol, 1 eq.) and $Pd(PPh_3)_4$ (84 mg, 79.7 µmol, 6 mol%) and degassed DMF (7 mL) that was degassed by 3 freeze-pump-thaw cycles were heated at 100°C and stirred in the dark overnight.

The mixture was cooled to room temperature, diluted with water (DMF mL), and the residue was filtered, washed with water and methanol and dried in vacuum to afford 857 mg of pure $\mathbf{f_1}$ with 91% yield. ^{1}H NMR (400 MHz, $CDCl_3$, δ): 7.87 (s, 2H), 7.73 (d, *J* = 8.3 Hz, 2H), 7.57 (s, 2H), 7.56 (s, 2H), 7.52 (s, 2H), 7.30 (dd, *J* = 8.4, 1.6 Hz, 2H), 2.77 (t, *J* = 7.6 Hz, 4H), 2.55 (s, 6H), 1.75-1.68 (m, 4H), 1.40-1.20 (s, 20H), 0.89 (t, *J* = 6.6 Hz, 6H). ^{13}C NMR (101 MHz, $CDCl_3$, δ): 142.60, 142.08, 139.54, 136.38, 133.77, 130.99, 130.07, 129.42, 127.86, 127.52, 125.14, 122.96, 120.24, 36.34, 32.04, 31.42, 29.65, 29.46, 29.41, 22.82, 16.35, 14.25. HRMS (MALDI HRMS) *m/z*: $[M]^+$ calcd for $C_{44}H_{52}S_4$, 764.3578; found,764.3558. TLC (Heptane/$CHCl_3$ 1:1): Rf = 66%.

2,5-bis-(6-decyl-3-methylthio-2-naphtyl)thieno[3,2-*b*]thiophene ($\mathbf{f_2}$): 94% yield from 3.70 g.
^{1}H NMR (400 MHz, $CDCl_3$, δ): 7.87 (s, 2H), 7.73 (d, *J* = 8.4 Hz, 2H), 7.57 (s, 2H), 7.56 (s, 2H), 7.52 (s, 2H), 7.30 (dd, *J* = 8.4, 1.7 Hz, 2H), 2.78 (t, *J* = 7.8 Hz, 4H), 2.55 (s, 6H), 1.75-1.68 (m, 4H), 1.40-1.20 (m, 28H), 0.89 (t, *J* = 7.2, 6.7 Hz, 6H). ^{13}C NMR (101 MHz, $CDCl_3$, δ): 142.60, 142.07, 139.54, 136.37, 133.77, 130.99, 130.07, 129.42, 127.86, 127.51, 125.14, 122.96, 120.23, 36.34, 32.05, 31.41, 29.77, 29.75, 29.69, 29.48, 29.45, 22.84, 16.34, 14.27. HRMS (MALDI HRMS) *m/z*: $[M]^+$ calcd for $C_{48}H_{60}S_4$, 708.2952; found, 708.2961. TLC (Heptane/$CHCl_3$ 1:1): Rf = 69%.

Typical procedure for the oxidation of 2,5-bis-(3-methylthio-2-naphtyl)thieno[3,2-*b*]thiophenes into sulfoxydes

2,5-bis-(6-octyl-3-methylsulfoxy-2-naphtyl)thieno[3,2-*b*]thiophene ($\mathbf{g_1}$): To a solution of $\mathbf{f_1}$ (857 mg, 1,21 mmol, 1 eq.) in $CHCl_3$ (stabilized with EtOH) at 0°C was added m-CPBA (556 mg, 2.42 mmol, 2.0 eq assuming 75% w/w in water) under air and the reaction was stirred at this temperature for 2h. Then the mixture was washed 3 times with aqueous Na_2CO_3 (5% w/w), dried over $MgSO_4$, filtered and evaporated to give 928 mg of crude $\mathbf{g_1}$ that was used in next step without further purification. ^{1}H NMR (400 MHz, $CDCl_3$, δ): 8.56 (s, 2H), 7.96 (d, *J* = 3.4 Hz, 2H), 7.85 (dd, *J* = 8.5, 2.9 Hz, 2H), 7.80 (s, 2H), 7.50-7.48 (m, 2H) ,7.48 (d, *J* = 1.5 Hz, 2H), 2.83 (t, *J* = 7.8, 7.3 Hz, 4H), 2.59 (d, *J* = 4.6 Hz, 6H), 1.77-1.69 (m, 4H), 1.40-1.20 (m, 20H), 0.89 (t, *J* = 7.0 Hz, 6H). ^{13}C NMR (101 MHz, $CDCl_3$, δ): 143.14, 141.99 and 141.97 (2 enantiomer couples), 141.40, 141.31, 140.28 and 140.25 (2 enantiomer couples), 133.26, 132.55, 130.81 and 130.78 (2 enantiomer couples), 130.21, 128.00, 127.19, 127.12, 124.48, 119.88, 42.54 and 42.50 (2 enantiomer couples), 36.26, 32.02, 31.33, 29.61, 29.39, 22.81, 14.24. HRMS (MALDI HRMS) *m/z*: $[M]^+$ calcd for $C_{44}H_{52}O_2S_4$, 796.3476; found, 796.3477. TLC (Toluene/EtOH 95:5): Rf = 33%.

2,5-bis-(6-decyl-3-methylsulfoxy-2-naphtyl)thieno[3,2-*b*]thiophene ($\mathbf{g_2}$): ^{1}H NMR (400 MHz, $CDCl_3$, δ): 8.56 (s, 2H), 7.96 (d, *J* = 3.4 Hz, 2H), 7.85 (dd, *J* = 8.5, 2.9 Hz, 2H), 7.80 (s, 2H), 7.51-7.46 (m, 2H), 7.48 (d, *J* = 1.5 Hz, 2H), 2.83 (t, *J* = 7.6 Hz, 4H), 2.59 (d, *J* = 4.6 Hz, 6H), 1.77-1.69 (m, 4H), 1.40-1.20 (m, 28H), 0.88 (t, *J* = 7.0 Hz, 6H). ^{13}C NMR (101 MHz, $CDCl_3$, δ): 143.14, 142.00 and 141.98 (2 enantiomer couples), 141.39 and 141.30 (2 enantiomer couples), 140.27 and 140.25 (2 enantiomer couples), 133.25, 132.55, 130.80 and 130.77 (2 enantiomer couples), 130.21, 127.99, 127.18, 127.12 and

127.10 (2 enantiomer couples), 124.47, 119.87, 42.53 and 42.50 (2 enantiomer couples), 36.25, 32.04, 31.33, 29.75, 29.73, 29.65, 29.46, 29.38, 22.82, 14.25. HRMS (MALDI HRMS) *m/z*: $[M]^+$ calcd for $C_{48}H_{60}O_2S_4$, 740.2850; found, 740.2855. TLC (Toluene/EtOH 95:5): Rf = 34%.

Typical procedure for the synthesis of 11 and 12

Synthesis of 11: To a suspension of P_2O_5 (86 mg, 605 μmol, 0.5 eq.) in trifluoromethanesulfonic acid (2.4 mL) at 0°C was added crude $\mathbf{g_1}$ (928 mg, 1.21 mmol, 1 eq.) and the reaction was stirred at room temperature for 64h. Then the mixture was added dropwise to a stirred ice/water mixture, the resulting precipitate was filtered, washed with water and refluxed in pyridine overnight. The mixture was cooled to room temperature, diluted with water and the precipitate was filtered and washed with water and methanol. The resulting residue was recrystallized in 1,2,4-trichlorobenzene to afford pure dioctyl-DN4T with 54% yield. Analytical grade **11** could be obtained by sublimation of the compound at 10^{-10} Pa at 380 °C. ^1H NMR (400 MHz, $C_2D_2Cl_4$, 120°C, δ): 8.34 (s, 2H), 8.32 (s, 2H), 7.98 (d, *J* = 8.5 Hz, 2H), 7.74 (s, 2H), 7.45 (d, *J* = 8.7 Hz, 2H), 2.90 (t, *J* = 7.6 Hz, 2H), 1.89-1.82 (m, 4H), 1.57-1.33 (m, 20H), 0.97 (t, *J* = 6.4 Hz, 3H). HRMS (MALDI HRMS) *m/z*: $[M]^+$ calcd for $C_{42}H_{44}S_4$, 676.2326; found, 676.2321.

Synthesis of 12: 59% yield from 3.75 mmol of $\mathbf{g_2}$. ^1H NMR (400 MHz, $C_2D_2Cl_4$, 120°C, δ): 8.34 (s, 2H), 8.32 (s, 2H), 7.98 (d, *J* = 8.5 Hz, 2H), 7.74 (s, 2H), 7.46 (d, *J* = 8.5 Hz, 2H), 2.90 (t, *J* = 7.7 Hz, 4H), 1.89-1.82 (m, 4H), 1.56-1.31 (m, 24H), 0.97 (t, *J* = 6.7 Hz, 6H). HRMS (MALDI HRMS) *m/z*: $[M]^+$ calcd for $C_{46}H_{52}S_4$, 732.2952; found, 732.2937.

Synthesis of **13**

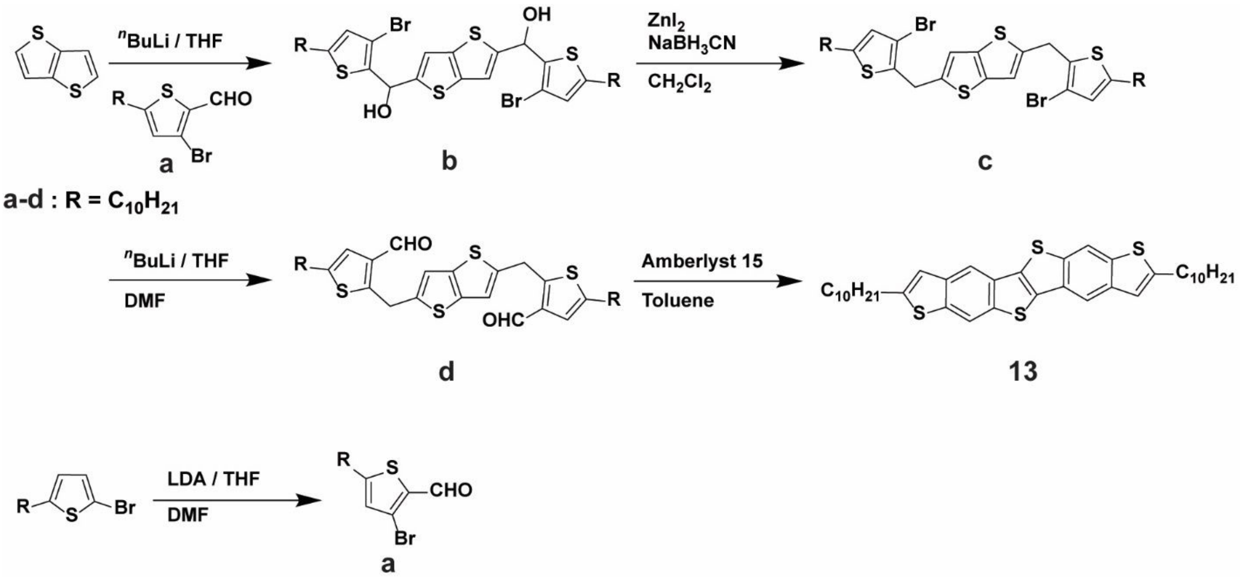

Scheme S1. Synthetic route of **13**.

Precursors **a** to **d** were synthesized following reported procedures[43]. To a stirred solution of the aldehyde **d** (0.71g, 1.06 mmol, 1.0 eq) in anhydrous Toluene (36 mL), Amberlyst

15 (0.636 g) was added under argon atmosphere. The mixture was stirred for reflux while water was removed using a Dean-Stark apparatus. After reaction for overnight, the reaction mixture was filtered while hot to remove Amberlyst 15 and then washed with hot toluene (about 30 mL). The filtrate was evaporated under vacuum to give the crude product as a pale yellow solid. The crude solid was purified by recrystallization with hot chlorobenzene to give the product as a white crystalline solid (60% yield). m.p. 573 K. IR (ATR-FTIR at room temperature, 4000 – 600 cm^{-1} range, peaks in cm^{-1}) 2957.54, 2915.99, 2848.55, 1467.69, 1426.77, 1322.21, 1276.99, 1253.08, 1132.42, 1006.47, 902.88, 871.78, 858.48, 830.20, 819.22, 784.30, 749.01, 719.49, 660.27. ^{1}H NMR (400 MHz, at 363 K, Toluene-d8) δ 7.83 (s, 2H), 7.75 (s, 2H), 6.78 (s, 2H), 2.70 (t, *J* = 7.5 Hz, 4H), 1.70–1.61 (m, 4H), 1.35–1.05 (m, 28H), 0.82 (t, *J* = 7.2 Hz, 6H). ^{13}C NMR (101 MHz, at 363 K, Toluene-d_8) δ 147.44, 139.35 (d), 138.22, 133.34, 131.86, 120.75, 117.42, 115.14, 32.46, 31.46, 31.43 (d), 30.22, 30.13, 29.94, 29.88, 29.74, 14.21. GC-MS *m/z*: $[M]^+$ calcd 632.26334; found 632.20. [M – one C_{10} side chain]$^+$ calcd 505.11466; found 505.05.

Crystal growth

Single crystals of all compounds were grown in different conditions. Single crystals of **1** and **3** were grown by the physical vapor transport (PVT) method[44]. Crystals of **1** were grown by PVT at atmospheric pressure in a high-purity argon flow of 0.1 l min^{-1} using 620 K for the sublimation zone and a growth duration of 75-100 minutes. Crystals of **3** were grown by PVT using 600 K for the sublimation zone at 10^{-10} bar in a 240 K m^{-1} temperature gradient. Crystals of **2**, **4** were grown by slow evaporation from a methanol solution at room temperature. Crystals of **5** were recrystallized from hexane at room temperature. Crystals of **6**-**8** were recrystallized from hot heptane. Crystals of **9**-**12** were recrystallized from hot toluene. Crystals of **13** were recrystallized from hot chlorobenzene.

Polarized optical microscopy coupled with hot stage

The cooperative transitions of all the crystals in this work were investigated using a hot stage Linkam THMS600 coupled with a liquid nitrogen cooling pump and mounted on a polarized optical microscope (POM) Nikon Eclipse 80i. The transitions were recorded with a Sony PXW-FS5 Mark II camera coupled with the POM with a LM Digital SLR Adapter with special anti-reflection coating, and plan achromatic optics with C-Mount 26 mm.

Differential scanning calorimetry

Differential scanning calorimetry (DSC) was carried out on a TA DSC-Q2000 and/or TA DSC-Q2500. Crystals (3-5 mg per sample) were analyzed in Tzero aluminum sealed pans and heated/cooled from room temperature to the selected temperatures at different heating and cooling rates (5-10 K min^{-1}) for two heating/cooling cycles centered around the phase transition temperatures for each compound.

For all compounds, we used the following notation to identify the different crystal polymorphs: the low-temperature forms are labelled as form I and the high-temperature forms are labelled as form II. In general, the DSC profiles often show multiple peaks, and they change shape, smoothen, and shift with each cycle as the result of crystallites fragmentation and consequent crystal size distribution decrease, and defects build up (*e.g.*, Figure S4). These signatures of thermal history are all typical features of

cooperative and shapeshifting polymorphic phase transitions that are highly dependent on heating/cooling cycles and rates, crystal size, and density of defects and dislocations[45].

Single crystal X-ray diffraction

All crystallographic measurements on crystals of **1** and **5** were made with monochromatic Cu radiation (λ = 1.54184 Å) using a Rigaku Synergy-i diffractometer coupled with an Oxford CryoSystems Cryostream 800 Series for variable-temperature analysis. Raw data processing utilized the program CrysAlisPro[46]. All structures were solved using direct methods and were refined against F^2 to convergence using all unique reflections and the program Shelxl-2018[47], as implemented within WinGX[48]. Selected crystallographic details and refinement parameters are given in Tables S2 and S3 and full crystallographic details in CIF format have been deposited with the CCDC reference numbers 2345569-2345572. Crystal structures of polymorphs of **2** (form I CCDC n. 1525484 and form II CCDC n. 1525485), polymorphs of **4** (form I CCDC n. 1567117 and form II CCDC n. 1567118), polymorphs of **6** (form I CCDC n. 2091417 and form II CCDC n. 679293), and low-temperature form of **10** (CCDC n. 2128319) were previously reported. Non-standard crystal structures of both low-temperature form and high-temperature form of **10** were refined from thin film data obtained by GIWAXS measurements and the details are available in the original publication (reference 29 in the main text). The characteristic temperature-activated structural order-disorder transition of the functional side chains of **5** and the consequent molecular reorientation of molecules within the lattice are depicted in figure S17 together with the main crystallographic changes between form I and form II.

Raman spectroscopy

Raman spectra were acquired using a custom-built Raman system. 785 nm diode laser (TOPTICA Photonics AG) was used for excitation at a power of 8 mW, focused on the sample using a 10x objective (Nikon). Two volume holographic notch filters (Ondax), each having OD>4 rejection with a spectral cut off ± 7 cm^{-1} around 785 nm, discard the Rayleigh-scattered light and allow measurements covering both Stokes and anti-Stokes sides of the spectrum. Low-temperature measurements were done inside an optical cryostat (Janis ST-500, USA). The sample was loaded in a special cell under helium flow, and the cell was directly loaded on the cryostat cold finger. Linkam TS1000EV high-temperature optical furnace to do above-room temperature measurements. High-temperature measurements were carried out under a continuous flow of nitrogen. The cooling/heating rate was kept constant at 5 K min^{-1}, with an equilibration time of 10 minutes for each temperature. The acquisition times for all samples were between 120-150 sec (8 accumulations). The averaged Raman spectra were obtained by adding the spectra acquired at three different angles of excitation polarization and adding them. In Figures S14-S16, temperature-dependent individual peak-shifting and broadening are depicted.

Density Functional Theory simulations and Hirshfeld surface analysis

Due to the size of our systems, we assume pairwise additivity, *i.e*. we assume that the energy of the system can be approximated as a sum of interaction energies between pairs of molecules:

$$W = \sum_{i} W_i + \sum_{i>j} E_{ij}$$

where W_i is the energy of the isolated molecule *i*, and E_{ij} is the interaction energy between the pair *i* and *j* taken in the absence of other molecules but in the geometry that they have in the whole system[49]. In the present study we approached the problem with the use of the CrystalExplorer suite[50]. The interaction energy is decomposed into a sum of the following terms,

$$E_{\text{tot}} = E_{\text{ele}} + E_{\text{pol}} + E_{\text{dis}} + E_{\text{rep}}$$

and explained in detail by Turner *et al*. in their implementation[51]. Monomer electron densities used to compute E_{ele}, E_{pol}, and E_{rep} were obtained at the B3LYP/6-31G(d,p) level of theory, and E_{dis} is the Grimme D2 dispersion correction, summed over all intermolecular atom pairs. Geometries were taken from crystallographic coordinates. Before starting the calculations, X–H distances of hydrogen atoms were restrained to the values obtained from neutron diffraction studies of small molecules. Lattice energies were also found with *CrystalExplorer*. This methodology has recently been used to rationalize the aggregation-induced-emission behavior of a series of aromatic thioethers[52]. The molecular electrostatic potential maps were obtained with Gaussian 09W[53], and visualized with the software MoleCoolQt[54]. Geometries were taken from crystallographic coordinates, with the X-H distances of hydrogen atoms restrained as stated above. The B3LYP functional and the aug-pcseg2 (superior to the aug-cc-pvtz) basis set optimized for DFT calculations[55] were used. We mapped the Hirshfeld surface over the shape index[56], and quantitatively analysed the most significant interactions. The shape index on the Hirshfeld surface allows to identify complementary, interacting regions of two molecular entities represented as hollows (red) and bumps (blue) as shown in the supplementary information.

Molecular Dynamics

We performed the simulations using the Large-scale Atomic/Molecular Massively Parallel Simulator, LAMMPS (30 Jul 2021)[57], with a timestep of 1 fs. We used the general AMBER force field (GAFF)[58] generated using the Antechamber tool and the AM1-BCC charge model. We validated the FF against experimental data by comparing the lattice parameters of a 2x2x2 supercell with the initial configuration of the CIF file deposited in CCDC after minimization (conjugate gradients) and thermalization of 300 ps in the isothermal-isobaric (NPT) ensemble at 100K or 255/300 K and 1 atm, using the Nose-Hoover thermostat and barostat[59]. The temperature and pressure were maintained with damping parameters of 100 fs and 1000 fs, respectively. We use PLUMED 2.7.1[60] to perform the post-processing of the results and qualitatively calculate the FES as a function of the CV to reflect distribution of the sample, under the assumption the system

is ergodic. We calculate the FES by $F(s)= -k_BT\ln P(s)$, where P(s) is the marginal distribution estimated by using likelihood maximization. The estimated error of the free energy is evaluated using block average analysis. The CV used for system **1** calculates the number of side chain dihedral angles (φ_1 and φ_2) that are within the range sampled at 100 K. In the case of system **6**, the initial conformation of the dihedral angles is different than the one at 100 K; therefore, a different CV is required. The CV used for system **6** calculates the average angle considering all the molecules in the system. For this purpose, φ_1 is mirrored to the positive range. The input files of this MD simulations are openly available on GitHub, at https://github.com/oreneli/DyanmicsPolymorphism.

Tables and Figures

Table S1. Transition temperatures, transition hysteresis, and shape change of the crystals' length (%) of all compounds.

	**Transition temperature range** (K)*	**Hysteresis** (K)**	**Shape change** (%)
**1**	251-255 h<br>236-240 c	15	-
**2**	164-180 h<br>155-165 c	9	6
**3**	486-520 h<br>307-329 c	179	5
**4**	215-219 h<br>213-216 c	2	3
**5**	242-247 h<br>237-242 c	5	1
**6**	190-226 h<br>109-149 c	81	12
**7**	224-251 h<br>130-155 c	94	10
**8**	248-266 h<br>163-194 c	85	15
**9**	388-393 h<br>378-382 c	10	5
**10**	386-391 h<br>380-385 c	6	9
**11**	452-457 h<br>435-440 c	17	5
**12**	446-452 h<br>435-442 c	11	4
**13**	326-340 h<br>310-326 c	16	10

* Data extracted from the second cycle of the DSC analysis. Transition temperatures are reporting for heating ramps (h) and cooling (c) separately. **Hysteresis was calculated from onset temperatures.

Table S2. Selected Crystallographic and Refinement Parameters of Form I at 100 K and Form II at 255 K of 1.

Empirical formula	$C_{26} H_{24} S_4$	$C_{26} H_{24} S_4$
Formula weight	464.69	464.69
Temperature	100(2) K	255(2) K
Wavelength	1.54184 Å	1.54184 Å
Crystal system	Triclinic	Triclinic
Space group	*P* -1	*P* -1
Unit cell dimensions	*a* = 6.0682(1) Å *α*= 91.751(1)°<br>*b* = 10.6927(2) Å *β*= 96.958(2)°<br>*c* = 17.4223(3) Å *γ* = 97.786(2)°	*a* = 6.2944(2) Å *α*= 94.024(2)°<br>*b* = 11.9761(4) Å *β*= 100.072(3)°<br>*c* = 16.0005(4) Å *γ* = 100.828(3)°
Volume	1110.51(3) $Å^3$	1159.78(6) $Å^3$
Z	2	2
Density (calculated)	1.390 Mg/m^3	1.331 Mg/m^3
Absorption coefficient	4.004 mm^{-1}	3.834 mm^{-1}
F(000)	488	488
Crystal size	0.31 x 0.08 x 0.03 mm^3	0.31 x 0.08 x 0.03 mm^3
Theta range for data collection	2.558 to 71.697°	2.821 to 71.709°
Reflections collected	22788	14007
Independent reflections	4300 [R(int) = 0.0383]	4433 [R(int) = 0.0450]
Completeness to theta = 70.000°	100.0 %	98.8 %
Absorption correction	Semi-empirical from equivalents	Semi-empirical from equivalents
Max. and min. transmission	1.00000 and 0.53143	1.00000 and 0.50659
Refinement method	Full-matrix least-squares on F^2	Full-matrix least-squares on F^2
Data / restraints / parameters	4300 / 0 / 277	4433 / 54 / 311
Goodness-of-fit on F^2	1.043	1.082
Final R indices [I>2sigma(I)]	R1 = 0.0347, wR2 = 0.0965	R1 = 0.0416, wR2 = 0.1181
R indices (all data)	R1 = 0.0369, wR2 = 0.0989	R1 = 0.0488, wR2 = 0.1251
Extinction coefficient	n/a	n/a
Largest diff. peak and hole	0.575 and -0.389 e.$Å^{-3}$	0.314 and -0.268 e $Å^{-3}$
CCDC n.	2345569	2345570

Table S3. Selected Crystallographic and Refinement Parameters of Form I at 100 K and Form II at 255 K of 5.

Empirical formula	C_{19} H_{30} Si	C_{19} H_{30} Si
Formula weight	286.52	286.52
Temperature	100(2) K	255(2) K
Wavelength	1.54184 Å	1.54184 Å
Crystal system	Orthorhombic	Orthorombic
Space group	*P* ca2_1	*P* nma
Unit cell dimensions	*a* = 21.5227(2) Å α= 90°<br>*b* = 15.5980(1) Å β= 90°<br>*c* = 11.3025(1) Å γ = 90°	*a* = 12.3528(2) Å α= 90°<br>*b* = 15.8375(3) Å β= 90°<br>*c* = 10.2187(2) Å γ = 90°
Volume	3794.37(5) Å^3	1999.16(6) Å^3
Z	8	4
Density (calculated)	1.003 Mg/m^3	0.952 Mg/m^3
Absorption coefficient	0.990 mm^{-1}	0.940 mm^{-1}
F(000)	1264	632
Crystal size	0.35 x 0.18 x 0.08 mm^3	0.35 x 0.18 x 0.08 mm^3
Theta range for data collection	2.833 to 71.482°	5.151 to 71.556°
Reflections collected	37192	15864
Independent reflections	6713 [R(int) = 0.0288]	2014 [R(int) = 0.0281]
Completeness to theta = 70.000°	100.0 %	100.0 %
Absorption correction	Semi-empirical from equivalents	Semi-empirical from equivalents
Max. and min. transmission	1.00000 and 0.45468	1.00000 and 0.48517
Refinement method	Full-matrix least-squares on F2	Full-matrix least-squares on F^2
Data / restraints / parameters	6713 / 1 / 380	2014 / 80 / 146
Goodness-of-fit on F^2	1.041	1.053
Final R indices [I>2sigma(I)]	R1 = 0.0297, wR2 = 0.0818	R1 = 0.0694, wR2 = 0.1988
R indices (all data)	R1 = 0.0306, wR2 = 0.0825	R1 = 0.0741, wR2 = 0.2049
Extinction coefficient	n/a	n/a
Largest diff. peak and hole	0.244 and -0.340 e.Å^{-3}	0.486 and -0.333 e.Å^{-3}
CCDC n.	2345571	2345572

Table S4. Interaction energy table (kJ/mol) for the dimers forming the crystal packing of compound 1 form I. The color chart is associated to that of Fig. S10.

	E_ele	E_pol	E_dis	E_rep	**E_tot**
	-6.8	-4.1	-70.5	39.8	**-40.9**
	-24.2	-3.7	-58.5	42.2	**-45.5**
	-0.8	-0.1	-6.9	3.0	**-4.7**
	-0.5	-0.2	-9.6	3.2	**-6.7**
	0.9	-0.5	-12.7	5.3	**-6.6**
	-10.7	-4.6	-75.8	49.5	**-42.1**

E_tot is the stabilization energy of the dimer.
Lattice energy: **-329.6** kJ/mol

Table S5. Interaction energy table (kJ/mol) for the dimers forming the crystal packing of compound 1 form II. The color chart is associated to that of Fig. S11.

	E_ele	E_pol	E_dis	E_rep	E_tot
(red)	-0.1	-0.1	-4.1	0.3	**-3.6**
(yellow)	-18.1	-3.3	-51.9	46.6	**-29.5**
(green)	0.9	-0.5	-10.5	4.0	**-5.6**
(aquamarine)	-7.8	-4.4	-71.7	40.8	**-42.3**
(blue)	-1.0	-0.2	-10.7	4.6	**-7.0**
(magenta)	-7.0	-4.0	-65.1	34.8	**-40.2**

E_tot is the stabilization energy of the dimer.
Lattice energy: **-288.0** kJ/mol

Table S6. Interaction energy table (kJ/mol) for the dimers forming the crystal packing of compound 6 form I. The color chart is associated to that of Fig. S12.

	E_ele	E_pol	E_dis	E_rep	E_tot
	-8.7	-0.8	-48.0	41.7	-25.9
	-17.1	-1.6	-79.9	62.0	-50.6
	-20.2	-3.6	-173.5	107.3	-108.9
	-1.5	-0.0	-10.3	6.5	-6.6
	-0.7	-0.0	-6.2	3.1	-4.2
	-0.6	-0.0	-4.4	2.8	-2.8
	-20.4	-3.7	-174.3	109.6	-108.5
	-0.3	-0.0	-3.0	1.1	-2.3
	-1.4	-0.0	-10.0	6.2	-6.4
	-8.7	-1.2	-49.3	42.4	-26.9
	-0.2	-0.0	-2.9	0.8	-2.2

E_tot is the stabilization energy of the dimer.
Lattice energy: **-403.1** kJ/mol

Table S7. Interaction energy table (kJ/mol) for the dimers forming the crystal packing of compound 6 form II. The color chart is associated to that of Fig. S13.

	E_ele	E_pol	E_dis	E_rep	E_tot
(red)	-11.9	-1.9	-88.0	51.1	-59.1
(yellow)	-13.1	-1.6	-62.7	46.7	-40.7
(green)	-1.0	-0.0	-8.0	5.3	-4.8
(blue)	-0.1	-0.0	-2.0	0.2	-1.6
(purple)	-0.5	-0.0	-3.9	2.2	-2.6

E_tot is the stabilization energy of the dimer.
Lattice energy: **-358.8** kJ/mol

Table S8. Lattice parameters of MD simulated compounds (1 and 6) and comparison to experimental value. Mean and Standard deviation were calculated based on the 100 data points of MD simulations after thermalization.

		a [Å]	b [Å]	c [Å]	α[°]	β[°]	γ[°]
**1** at 100K	Mean	6.143086	10.49037	17.30478	92.4045	96.26015	97.90969
	σ	0.063571	0.098349	0.116741	0.342481	0.357015	0.342501
	Exp[a]	6.0682	10.6927	17.4223	91.751	96.958	97.786
	Error	1%	-2%	-1%	1%	-1%	0%
**1** at 300K	Mean	6.31992	13.872	13.89596	100.4139	101.6591	100.6499
	σ	0.02579	0.074246	0.078162	0.561955	0.764885	0.75835
	Exp[b]	6.2944	11.9761	16.0005	94.024	100.072	100.828
	Error	0%	16%	-13%	7%	2%	0%
**6** at 100K	Mean	8.454745	5.28708	27.67161	93.07	92.69	87.39
	σ	0.065152	0.041325	0.138259	0.244357	0.385471	0.290042
	Exp[c]	8.1001	5.6595	27.2931	93.776	93.815	92.932
	Error	4%	-7%	1%	-1%	-1%	-6%
**6** at 300K	Mean	5.509873	8.6934	28.24503	89.97	92.90	90.01
	σ	0.020478	0.0329	0.066528	0.532576	0.200737	0.20275
	Exp[d]	5.927	7.88	29.18	90	92.443	90
	Error	-7%	10%	-3%	0%	0%	0%

[a] See Table S2
[b] See Table S2
[c] CCDC 2091417
[d] CCDC 679293

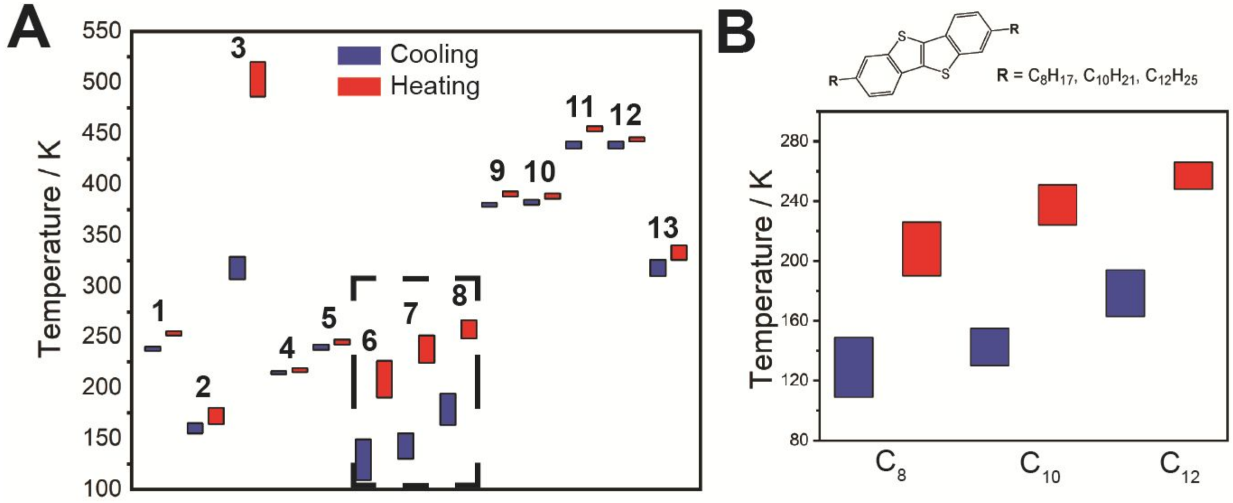

Fig. S1.

(A) Heating and cooling transition windows for compounds **1**-**13**. The dashed area highlights the transition temperatures of compounds **6**-**8** that are shown in detail in panel (B).

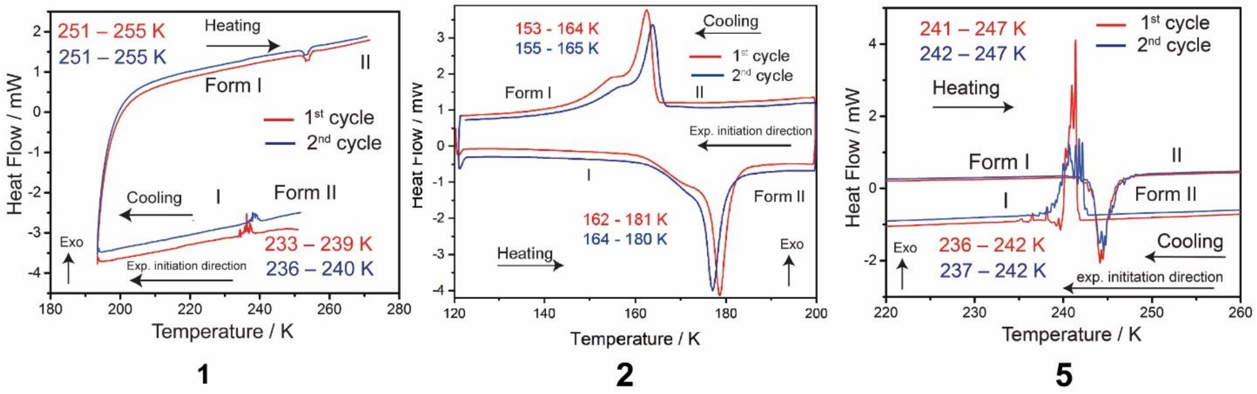

Fig. S2.

From left to right, DSCs of **1**, **2**, and **5**.

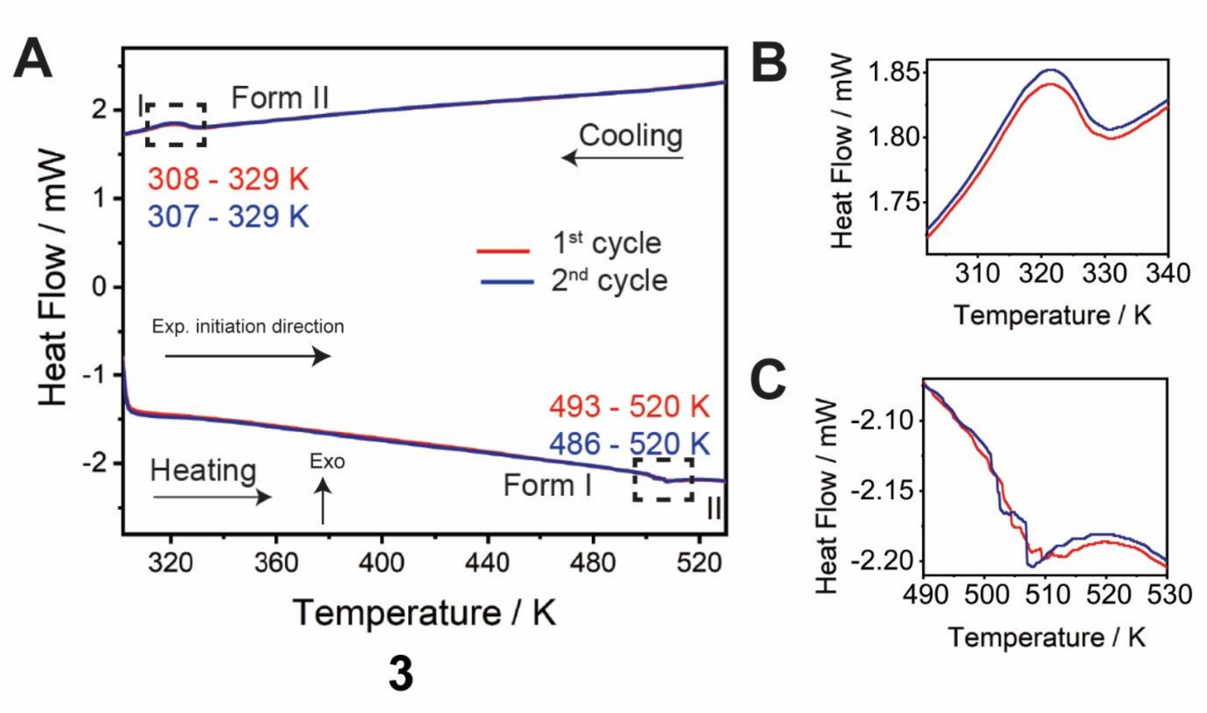

Fig. S3.

(A) DSC of **3**. The dotted boxes highlight the reversible cooperative transitions. The transition peaks are magnified in panel (B) in the cooling ramp and panel (C) in the heating ramp.

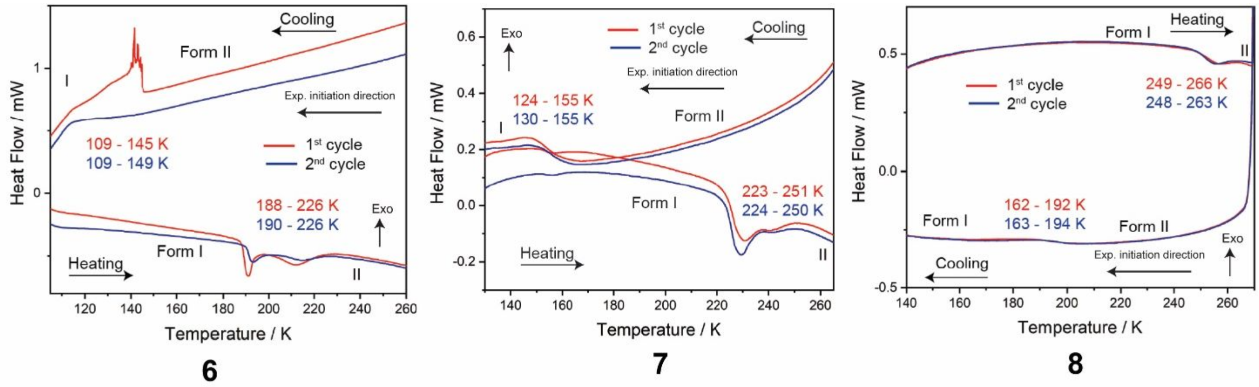

Fig. S4.

From left to right, DSCs of **6**, **7**, and **8**.

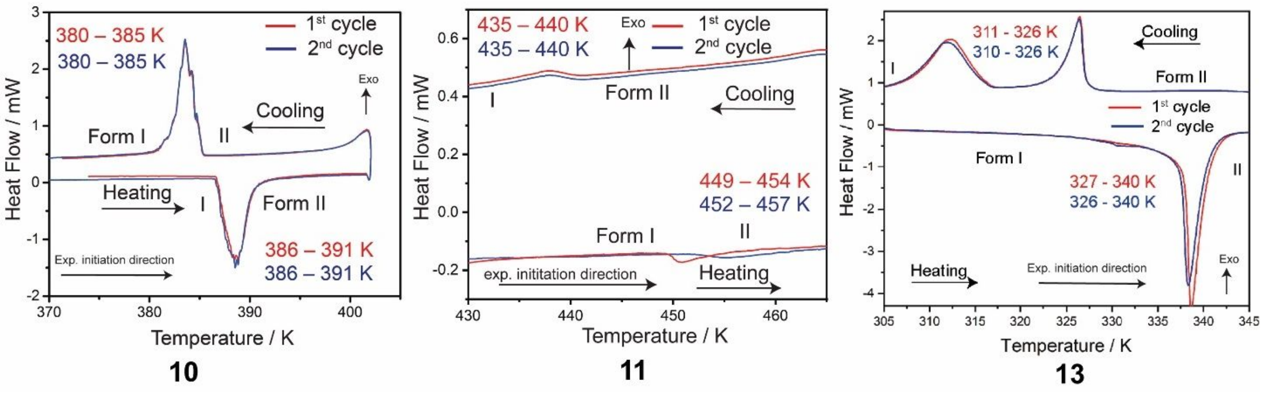

Fig. S5.

From left to right, DSCs of **10**, **11**, and **13**.

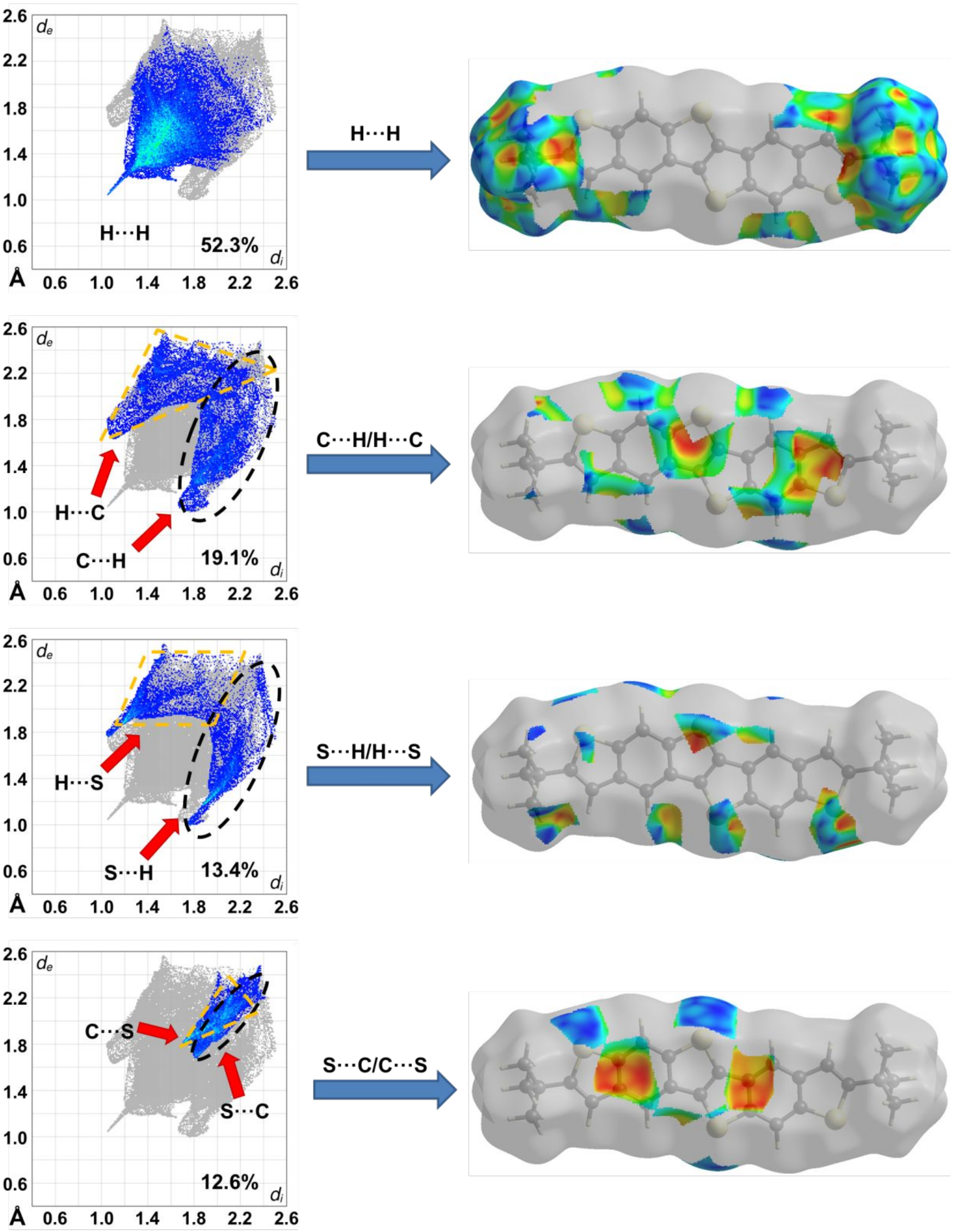

Fig. S6.

Fingerprint plots of compound **1**, form I resolved into different interactions showing the percentages of contacts contributing to the total Hirshfeld surface area of the molecule. Surfaces in the right-hand columns highlight the relevant surface patches associated with the specific contacts in the total Hirshfeld surface area of compound **1**, form I.

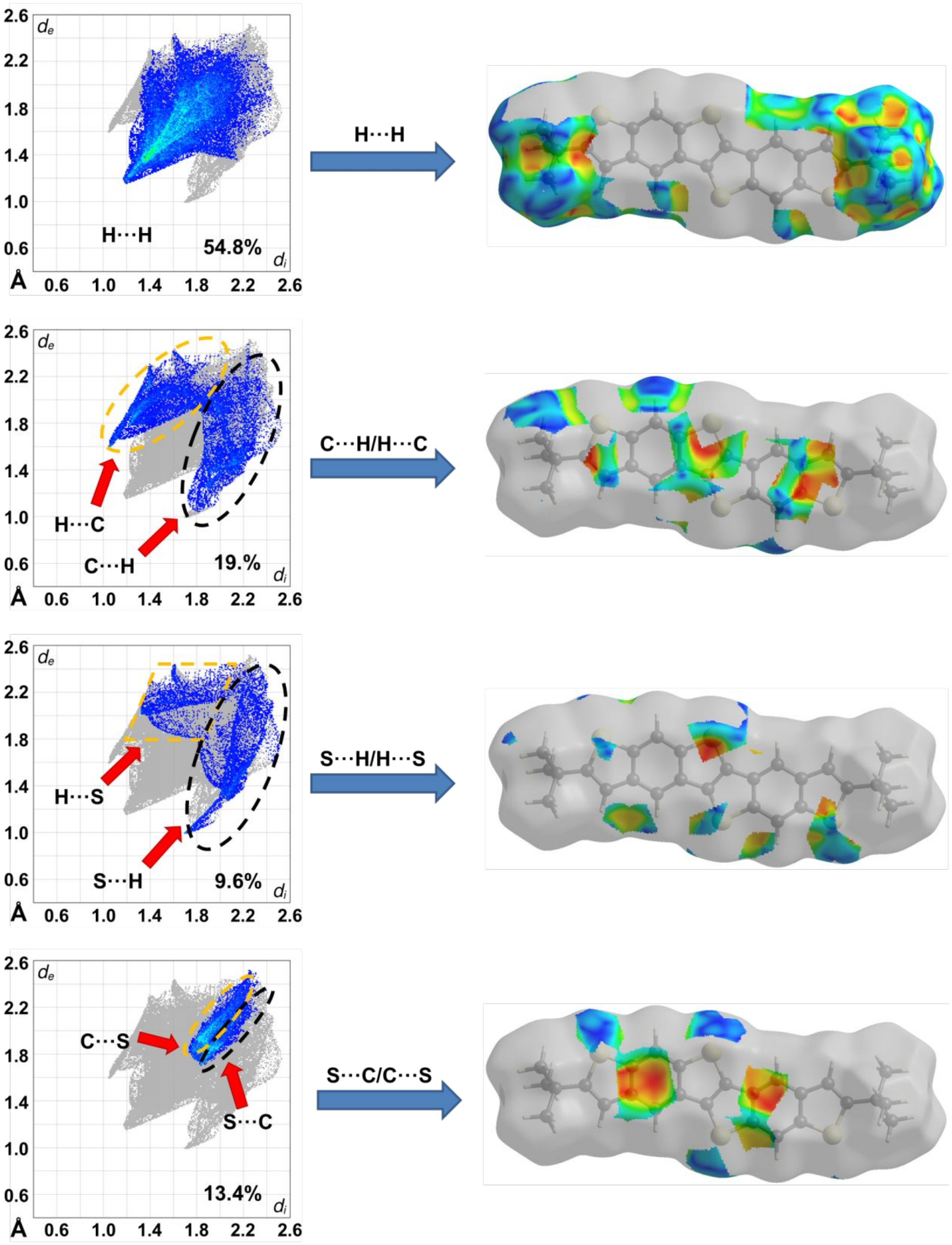

Fig. S7.

Fingerprint plots of compound **1**, form **II** resolved into different interactions showing the percentages of contacts contributing to the total Hirshfeld surface area of the molecule. Surfaces in the right-hand columns highlight the relevant surface patches associated with the specific contacts in the total Hirshfeld surface area of compound **1**, form **II**.

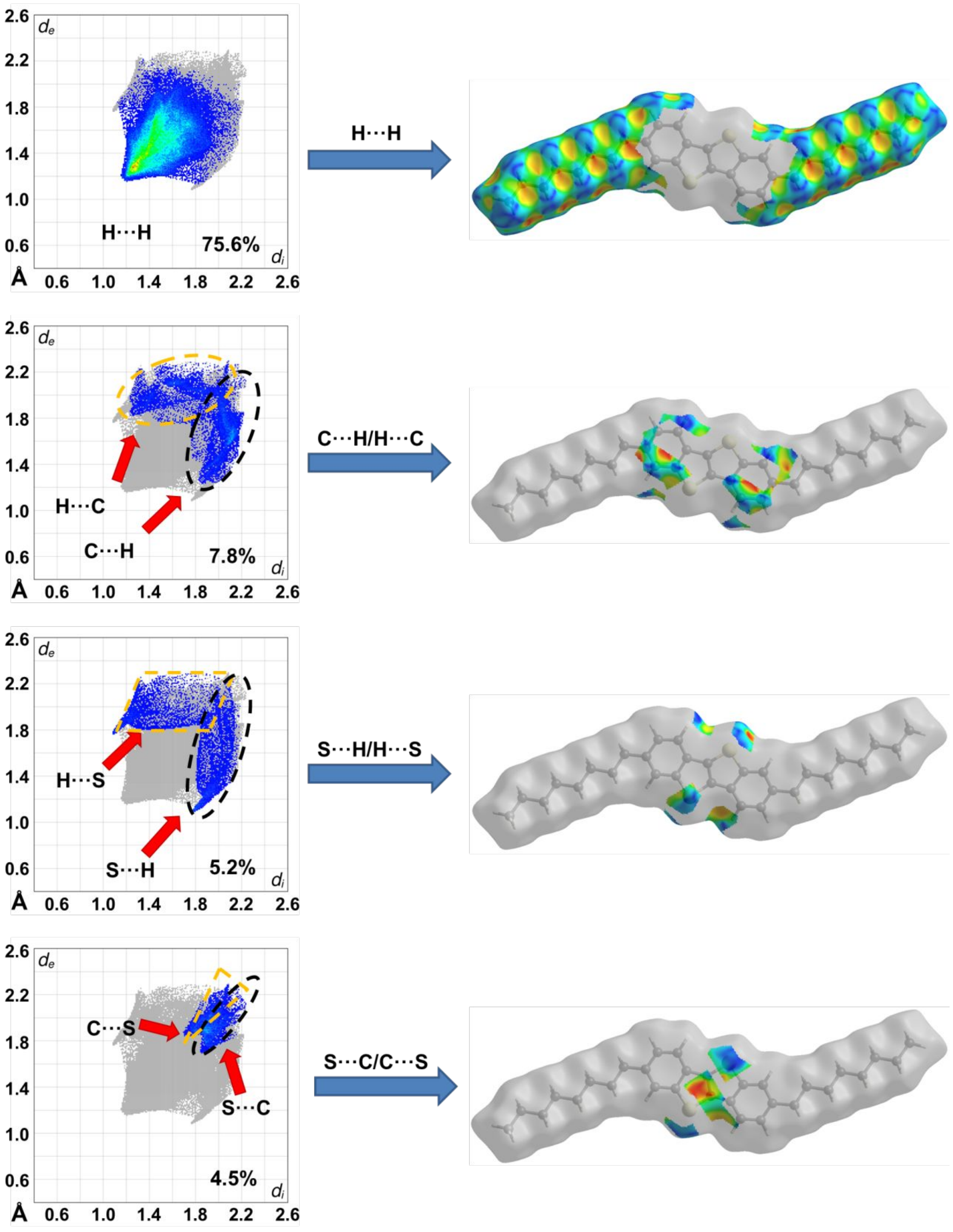

Fig. S8.

Fingerprint plots of compound **6**, form I resolved into different interactions showing the percentages of contacts contributing to the total Hirshfeld surface area of the molecule. Surfaces in the right-hand columns highlight the relevant surface patches associated with the specific contacts in the total Hirshfeld surface area of compound **6**, form I.

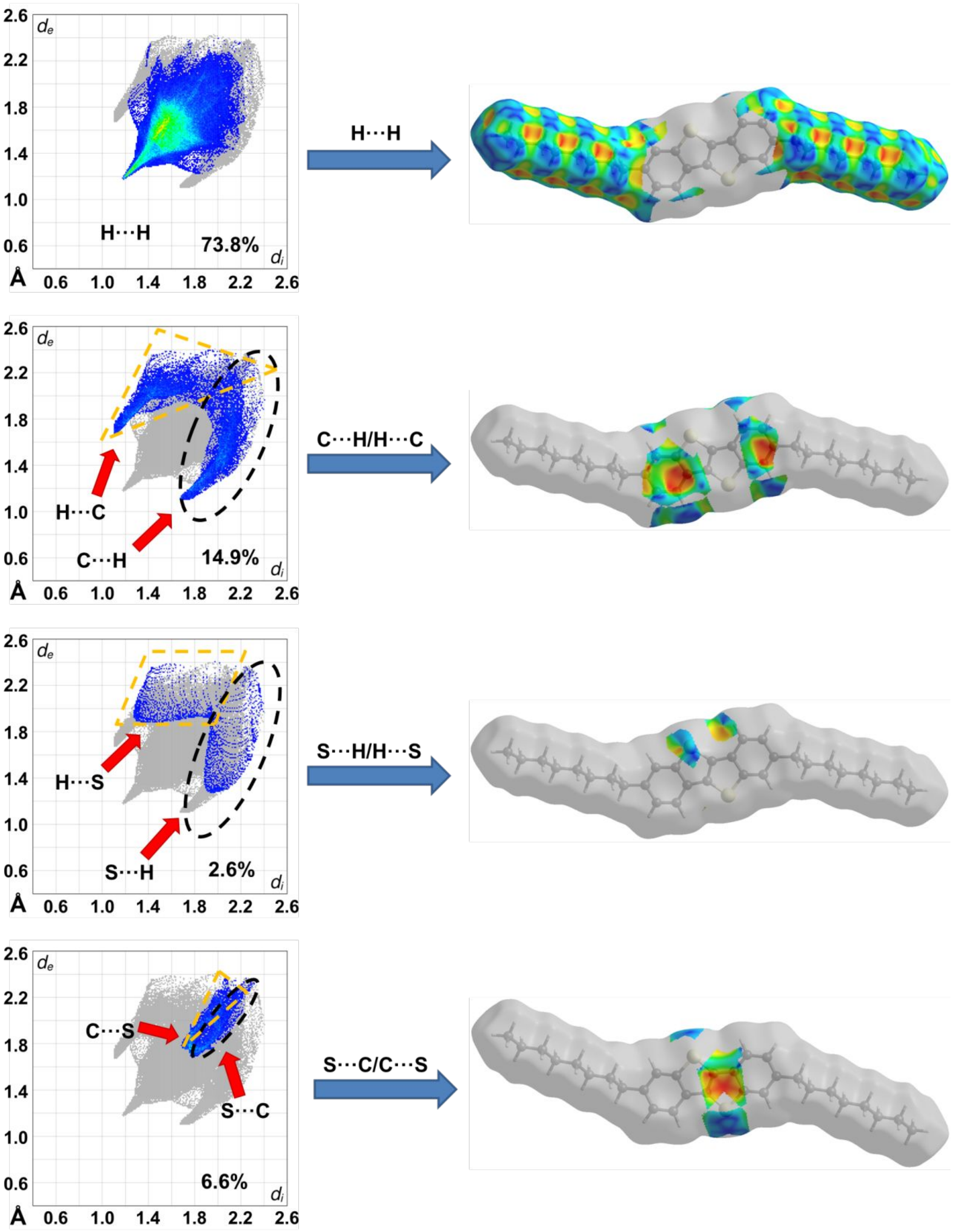

Fig. S9.

Fingerprint plots of compound **6**, form **II** resolved into different interactions showing the percentages of contacts contributing to the total Hirshfeld surface area of the molecule. Surfaces in the right-hand columns highlight the relevant surface patches associated with the specific contacts in the total Hirshfeld surface area of compound **6**, form **II**.

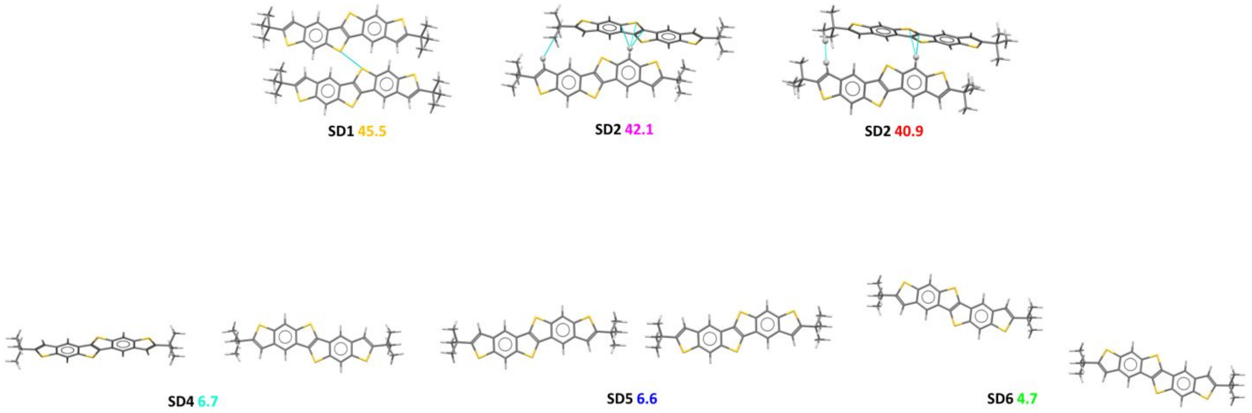

Fig. S10.

Supramolecular hierarchy of the five most stabilizing interactions in compound **1** form I as provided by CrystalExplorer. Blue lines represent short intermolecular contacts below the sum of the van der Waals radii of the respective atoms. Color chart: grey = C, white = H, yellow = S.

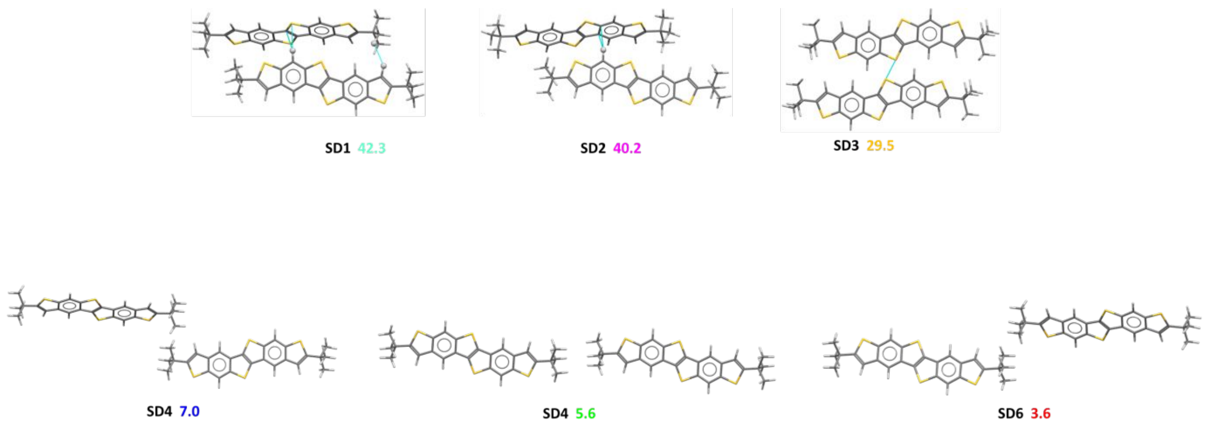

Fig. S11.

Supramolecular hierarchy of the five most stabilizing interactions in compound **1** form II as provided by CrystalExplorer. Blue lines represent short intermolecular contacts below the sum of the van der Waals radii of the respective atoms. Color chart: grey = C, white = H, yellow = S.

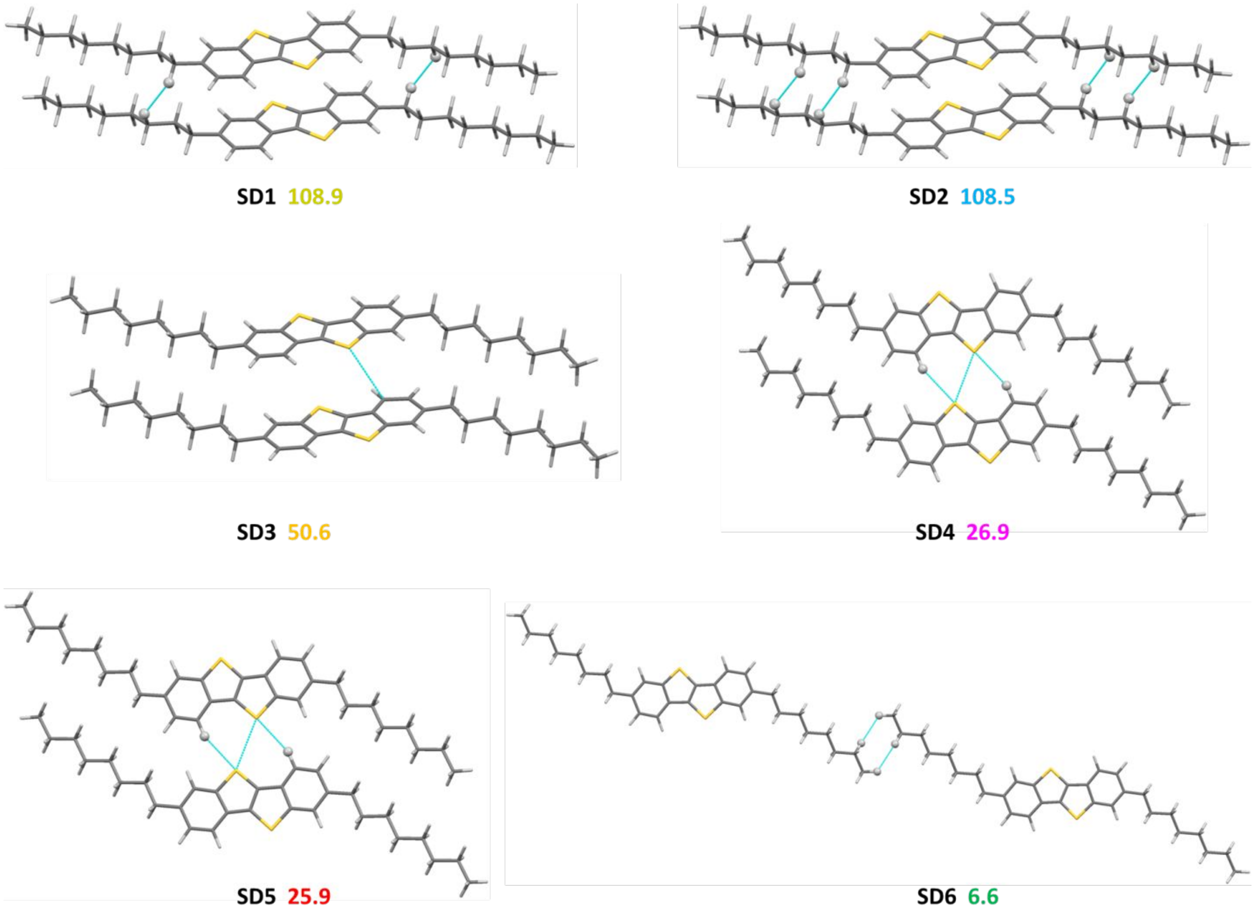

Fig. S12.

Supramolecular hierarchy of the six most stabilizing interactions in compound **6** form I as provided by CrystalExplorer. Blue lines represent short intermolecular contacts below the sum of the van der Waals radii of the respective atoms. Color chart: grey = C, white = H, yellow = S.

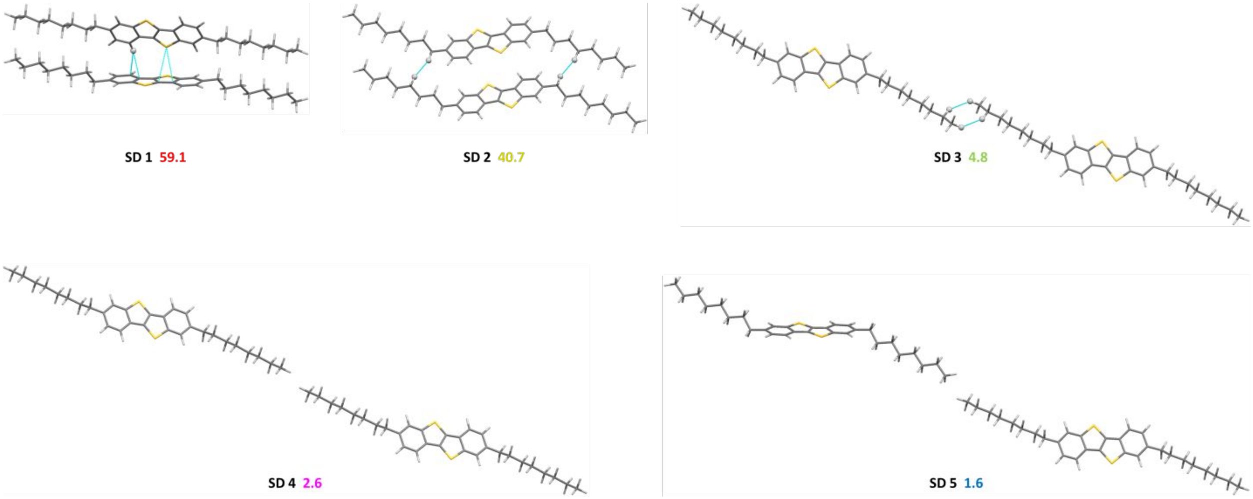

Fig. S13.

Supramolecular hierarchy of the five most stabilizing interactions in compound **6** form II as provided by CrystalExplorer. Blue lines represent short intermolecular contacts below the sum of the van der Waals radii of the respective atoms. Color chart: grey = C, white = H, yellow = S.

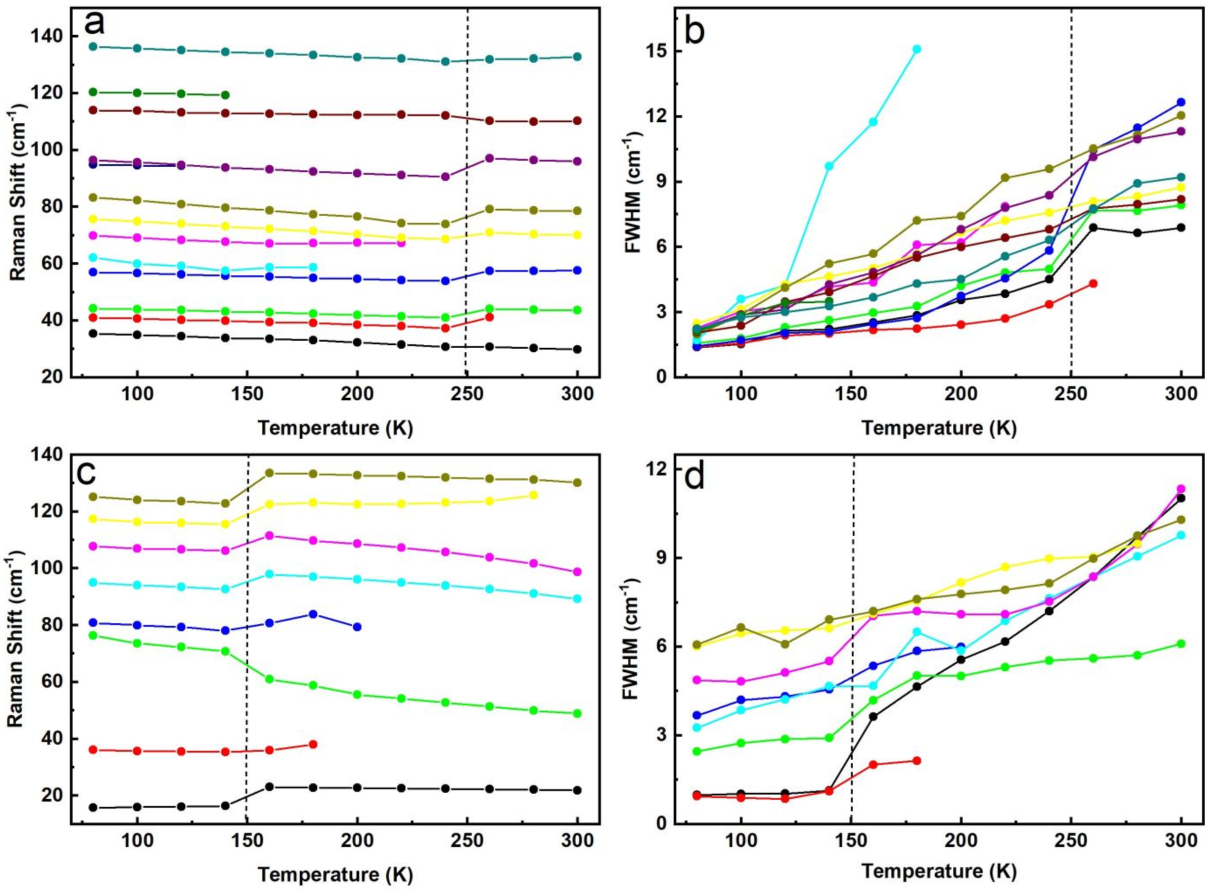

Fig. S14.

Temperature dependence of Raman peak-shift (a, c) and broadening (b, d) for samples **1** (a, b) and **6** (c,d) corresponding to the Raman spectra in Fig. 3 (P,Q) of the main text. The dotted vertical line indicates the polymorphic phase transition temperatures.

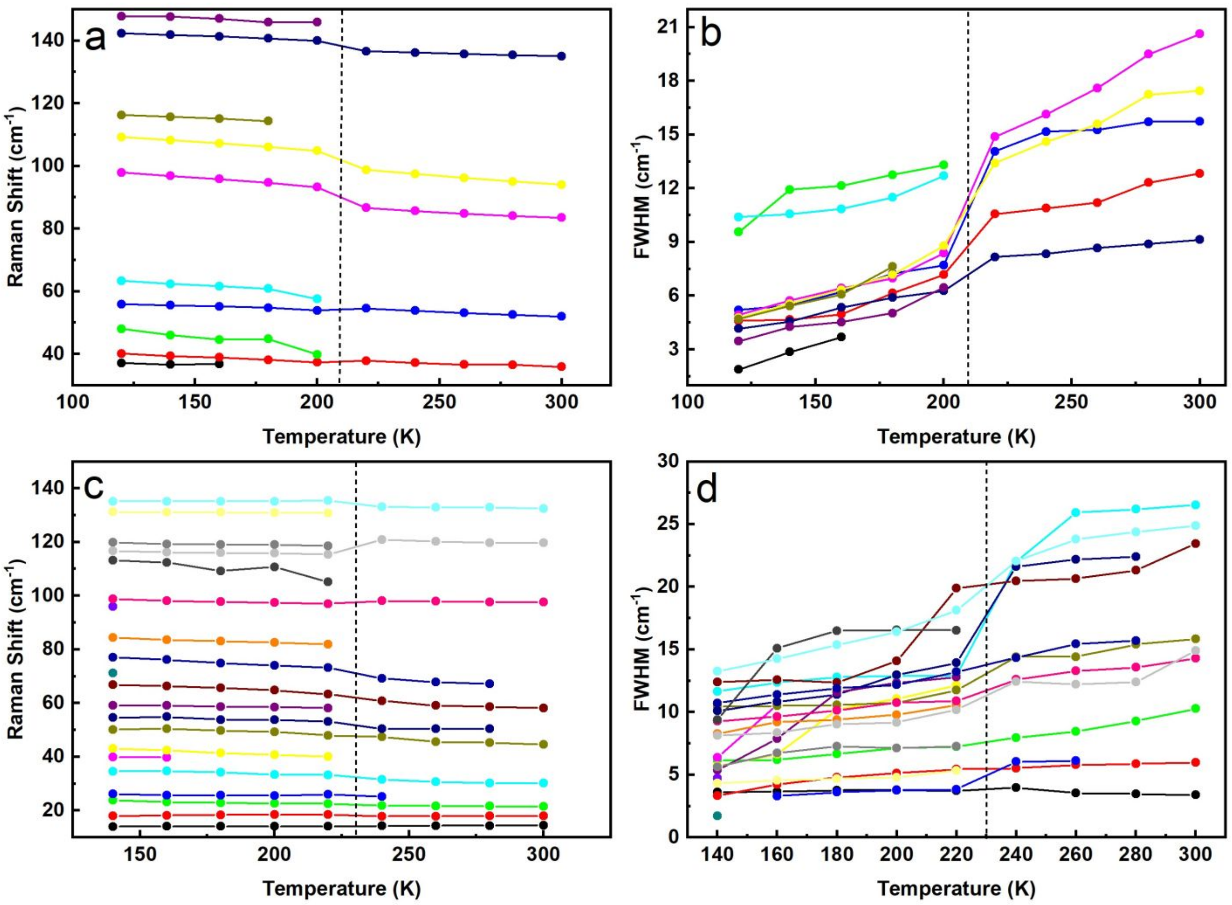

Fig. S15.

Temperature dependence of Raman peak-shift (a, c) and broadening (b, d) for samples **4** (a, b) and **5** (c,d) corresponding to the Raman spectra in Fig. 4 (A,B) of the main text. The dotted vertical line indicates the polymorphic phase transition temperatures.

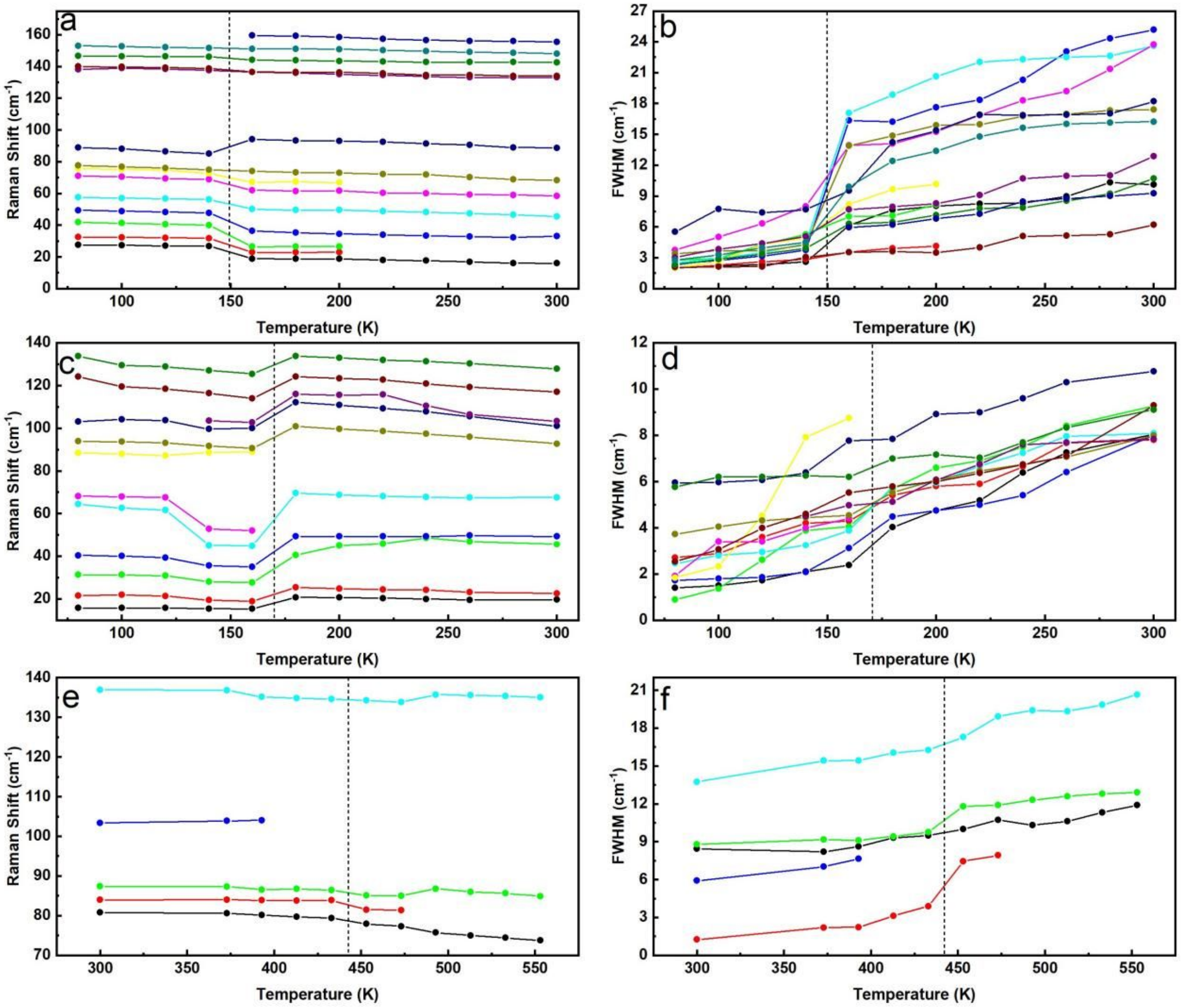

Fig. S16.

Temperature dependence of Raman peak-shift (a, c, e) and broadening (b, d, f) for samples **2** (a, b), **7** (c, d) and **11** (e, f) corresponding to the Raman spectra in Fig. 4 (C) of the main text. The dotted vertical line indicates the polymorphic phase transition temperature.

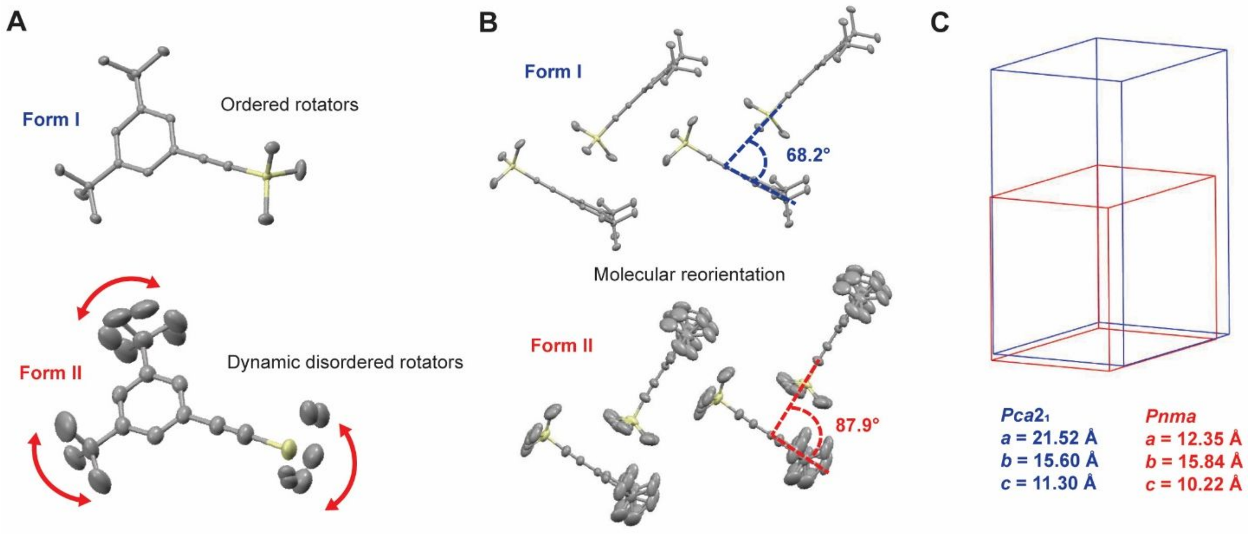

Fig. S17.

(A) Ordered and disordered side chains of form I (top) and form II (bottom) of **5**, respectively. (B) Molecular reorientation of the aromatic cores within the herringbone packing from form I (top) to form II (bottom) of **5**. (C) Overlapping unit cells of form I (blue) and form II (red) of **1** with the changes in space group and unit cell highlighted. Note that the *a* axis is doubling from form II to form I due to symmetry constraints.

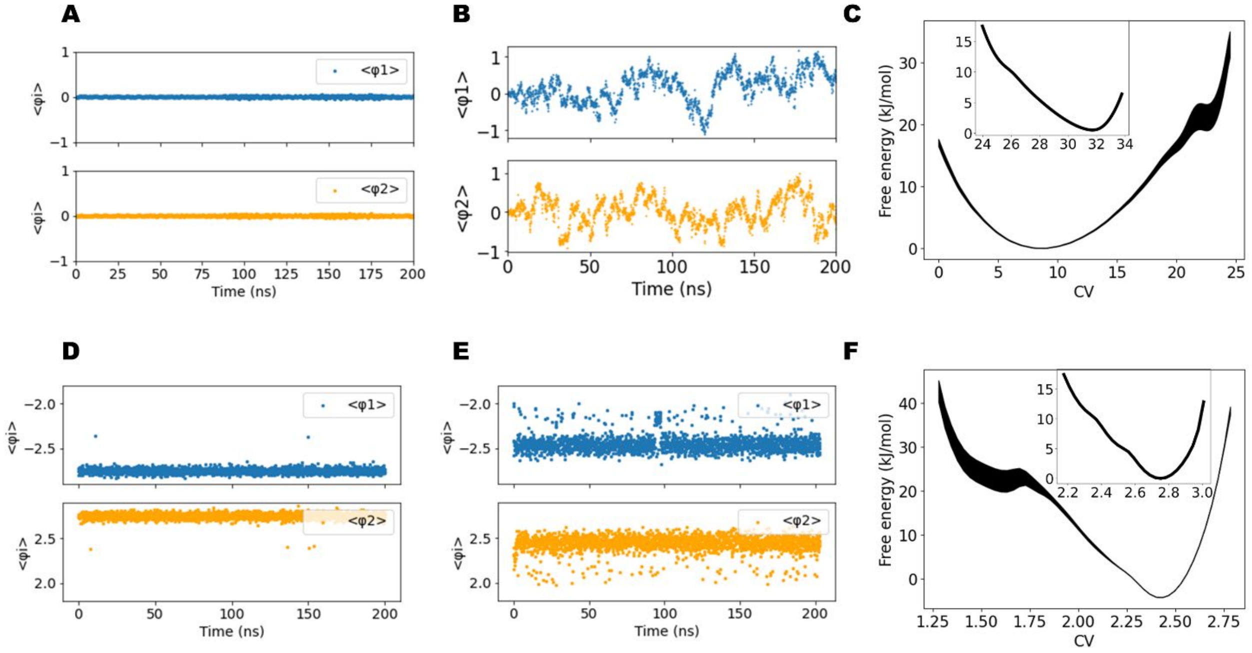

Fig. S18.
<φ_1> and <φ_2> fluctuations in system **1,** when using 4x4x4 supercell. (A) at 100 K (B) and 255 K (C) Free energy surface of **1** as function of a CV at 100 K (inset) and at 255 K. <φ_1> and <φ_2> fluctuations in system **6,** when using 4x4x4 supercell, (D) at 100 K (E) and 300 K, and (F) Free energy surface of **6** as function of a CV at 100 K (inset) and 300 K.

Supporting references:

39. Richard A. *Conception, synthèse et caractérisation de semi-conducteurs moléculaires à dimensionnalité élevée*. PhD Thesis, Université libre de Bruxelles, 2017. difusion.ulb.ac.be/vufind/Record/ULB-DIPOT:oai:dipot.ulb.ac.be:2013/256602/Holdings
40. Valandro, S.R.; He, R., Bullock, J. D.; Arman, H.; Schanze, K. S. Ultrafast Excited-State Dynamics in trans-(N-Heterocyclic carbene)platinum(II) Acetylide Complexes. *Inorg. Chem.* **2021**, *60*, 10065–10074. DOI : 10.1021/acs.inorgchem.1c00288
41. Mitsui, C.; Annaka, T.; Nakamura, K.; Mitani, M.; Hashizume, D.; Nakahara, Yamagishi, M.; Takeya, J.; Okamoto, T. Alkylated oxygen-bridged V-shaped molecules: impacts of the substitution position and length of the alkyl chains on the crystal structures and fundamental properties in aggregated forms. *Polym. J.* **2017**, *49*, 215–221. DOI: 10.1038/pj.2016.105
42. Niimi, K.; Kang, M. J.; Miyazaki, E.; Osaka, I.; Takimiya, K. General Synthesis of Dinaphtho[2,3-b:2′,3′-f]thieno[3,2-b]thiophene (DNTT) Derivatives. *Org. Lett*. **2011**, *13*, 3430–3433. DOI: 10.1021/ol2010837
43. Chen, H. Y.; Schweicher, G.; Planells, M.; Ryno, S. M.; Broch, K.; White, A. J. P.; Simatos, D.; Little, M.; Jellett, C.; Cryer, S. J.; Marks, A.; Hurhangee, M.; Brédas, J. L.; Sirringhaus, H.; McCulloch, I. Crystal Engineering of Dibenzothiophenothieno[3,2-b]thiophene (DBTTT) Isomers for Organic Field-Effect Transistors. **2018**, *30*, 7587–7592. DOI: 10.1021/acs.chemmater.8b02757
44. Jiang, H.; Kloc, C. Single-crystal growth of organic semiconductors. *MRS Bulletin* **2013**, *38*, 28–33. DOI: 10.1557/mrs.2012.308
45. Sahoo, S. C.; Panda, M. K.; Nath, N. K.; Naumov, P. Biomimetic Crystalline Actuators: Structure–Kinematic Aspects of the Self-Actuation and Motility of Thermosalient Crystals. *J. Am. Chem. Soc.* **2013**, *135*, 12241–12251. DOI: 10.1021/ja404192g
46. Rigaku Oxford Diffraction, CrysAlisPro (Rigaku Oxford Diffraction, 2016).
47. Sheldrick, G. M. Crystal structure refinement with SHELXL. *Acta Cryst. C*, **2015**, *71*, 3–8. DOI: 10.1107/S2053229614024218
48. Farrugia, L. J. WinGX and ORTEP for Windows: an update. *J. App. Cryst.* **2012**, *45*, 849–854. DOI: 10.1107/S0021889812029111
49. Stone, A. J. Non-Covalent Interactions in Quantum Chemistry and Physics, 3-26, Elsevier, A. Otero de la Roza, G.A. DiLabio Eds., 2017. DOI: 10.1016/B978-0-12-809835-6.00002-5
50. Turner, M. J.; McKinnon, J. J.; Wolff, S. K.; Grimwood, D. J.; Spackman, P. R.; Jayatilaka, D.; Spackman, M. A. *CrystalExplorer17*, University of Western Australia, 2017.
51. Turner, M. J.; Grabowsky, S.; Jayatilaka, D.; Spackman, M. A. Accurate and Efficient Model Energies for Exploring Intermolecular Interactions in Molecular Crystals. *J. Phys. Chem. Lett.* **2014**, *5*, 4249–4255. DOI: 10.1021/jz502271c
52. Saccone, M.; Riebe, S.; Stelzer, J.; Wölper, C; Daniliuc, C. G.; Voskuhl, J.; Giese, M. Structure–property relationships in aromatic thioethers featuring aggregation-induced emission: solid-state structures and theoretical analysis. *CrystEngComm* **2019**, *21*, 3097–3105. DOI: 10.1039/C9CE00444K

53. Frisch, M. J.; Trucks, G. W.; Schlegel, H. B.; Scuseria, G. E.; Robb, M. A.; Cheeseman, J. R.; Scalamani, G.; Barone, V.; Mennucci, B.; Peterson, G. A.; Nakatsuji, H.; Caricato, M.; Li, X.; Hratchian, H. P.; Izmaylov, A. F.; Bloino, J.; Zheng, G.; Sonnenberg, J. L.; Hada, M.; Ehara, M.; Toyota, K.; Fukuda, T. R.; Hasegawa, J.; Ishida, M.; Nakajima, T.; Honda, Y.; Kitao, O.; Nakai, H.; Vreven, T.; Throssell, K.; Montgomery, J. A.; Peralta, Jr.J. A.; Ogliaro, F.; Bearpark, M.; Heyd, J. J.; Brothers, E.; Kudin, K. N.; Staroverov, V. N.; Kobayashi, R.; Normand, J.; Raghacachari, K.; Rendell, A.; Burant, J. C.; Iyengar, S. S.; Tomasi, Cossi, M.; Rega, N.; Millam, J. M.; Klene, M.; Knox, J. E.; Cross, J. B.; Bakken, V.; Adamo, C.; Jaramillo, J.; Stratmann, R. E.; Gomperts, R.; Yazyev, O.; Austin, A. J.; Cammi, R.; Pomelli, C.; Ochterski, J. W.; Martin, R. L.; Morokuma, K.; Zakrzewski, V. G.; Voth, G. A.; Salvador, P.; Dannenberg, J. J.; Dapprich, S.; Daniels, A. D.; Farkas, Ö.; Foresman, J. B.; Ortiz, J. V.; Cioslowski, J.; Fox, D. J. *Gaussian 09 Revision C.01*, Gaussian, Inc.: Wallingford, CT, 2009.
54. Hübschle, C.B.; Dittrich, B. MoleCoolQt – a molecule viewer for charge-density research. *J. Appl. Crystallogr*. **2012**, *44*, 238–240. DOI: 10.1107/S0021889810042482
55. Jensen, F. Unifying General and Segmented Contracted Basis Sets. Segmented Polarization Consistent Basis Sets. *J. Chem. Theory Comput*. **2014**, *10*, 1074–1085. DOI: 10.1021/ct401026a
56. Spackman, M. A.; Jayatilaka, D. Hirshfeld surface analysis. *CrystEngComm*, **2009**, *11*, 19–32. DOI: 10.1039/B818330A
57. Thompson, A. P.; Aktulga, H. M.; Berger, R.; Bolintineanu, D. S.; Brown, W. M.; Crozier, P.M.; in 't Veld, P. J.; Kohlmeyer, A.; Moore, S. G.; Nguyen, T. D.; Shan, R.; Stevens, M. J.; Tranchida, J.; Trott, C.; Plimpton, S.J. LAMMPS - a flexible simulation tool for particle-based materials modeling at the atomic, meso, and continuum scales. *Comput. Phys. Commun.* **2022**, *271*, 108171. DOI: 10.1016/j.cpc.2021.108171
58. Wang, J.; Wolf, R. M.; Caldwell, J. W.; Kollman, P. A.; Case, D. A. Development and testing of a general amber force field. *J. Comput. Chem.* **2004**, *25*, 1157–1174. DOI: 10.1002/jcc.20035
59. Shinoda, W.; Shiga, M.; Mikami, M. Rapid estimation of elastic constants by molecular dynamics simulation under constant stress. *Phys. Rev. B*, **2004**, *69*, 134103. DOI: 10.1103/PhysRevB.69.134103
60. Tribello, G. A.; Bonomi, M.; Branduardi, D.; Camilloni, C.; Bussi, G. PLUMED 2: New feathers for an old bird. *Comput. Phys. Commun.* **2014**, *185*, 604-613. DOI: 10.1016/j.cpc.2013.09.018
